# Finite free convolutions of polynomials

**DOI:** 10.1007/s00440-021-01105-w

**Published:** 2022-02-18

**Authors:** Adam W. Marcus, Daniel A. Spielman, Nikhil Srivastava

**Affiliations:** 1 École Polytechnique Fédérale de Lausanne, Lausanne, Switzerland; 2 Yale University, New Haven, CT USA; 3 UC Berkeley, Berkeley, CA USA

**Keywords:** Free probability, Random matrix theory, Interlacing families, Ramanujan graphs, 60B20 (random matrices), 46L54 (free probability), 05E99 (algebraic combinatorics), 26C10 (polynomials, location of zeros)

## Abstract

We study three convolutions of polynomials in the context of free probability theory. We prove that these convolutions can be written as the expected characteristic polynomials of sums and products of unitarily invariant random matrices. The symmetric additive and multiplicative convolutions were introduced by Walsh and Szegö in different contexts, and have been studied for a century. The asymmetric additive convolution, and the connection of all of them with random matrices, is new. By developing the analogy with free probability, we prove that these convolutions produce real rooted polynomials and provide strong bounds on the locations of the roots of these polynomials.

## Introduction

We study three convolutions on polynomials that are inspired by free probability theory. Instead of capturing the limiting spectral distributions of ensembles of random matrices, we show that these capture the expected characteristic polynomials of random matrices in a fixed dimension. We develop the analogy with Free Probability by proving that Voiculescu’s *R* and *S*-transforms can be used to prove upper bounds on the extreme roots of these polynomials. Two of the convolutions have been classically studied. The third, and the connection of all of them with random matrices, is new. We begin by defining the three convolutions and stating the algebraic identities that establish this connection, as well as basic results regarding their real-rootedness properties.

### Algebraic identities and real rootedness

#### Symmetric additive convolution

##### Definition 1.1

For complex univariate polynomials

$$\begin{aligned} p (x) = \sum _{i=0}^{d} x^{d-i} (-1)^{i} a_{i} \quad \text { and} \quad q (x) = \sum _{i=0}^{d} x^{d-i} (-1)^{i} b_{i} \end{aligned}$$

of degree at most *d*, the *dth symmetric additive convolution* of *p* and *q* is defined as:1

where *D* denotes differentiation with respect to *x*. Note that we have defined a *sequence* of convolutions parameterized by *d*, and in general , even if both *p* and *q* have degree less than *c* and *d*; we will discuss this point more in Sect. 1.4.

Observe that the definition above has the compact form:2where ${\hat{p}}$ and ${\hat{q}}$ are the unique polynomials satisfying ${\hat{p}}(D)x^d=p(x)$ and ${\hat{q}}(D)x^d=q(x)$. This reveals that  is symmetric, bilinear in its arguments, and commutes with differentiation and translation; i.e.,Note that the identity element for  is the polynomial $x^d$.

For a square $d\times d$ complex matrix *M*, we define

$$\begin{aligned} \chi _{x} \left( M \right) {\mathop {=}\limits ^{\mathrm {def}}}\det (xI - M), \end{aligned}$$

its characteristic polynomial in the variable *x*. We show that for monic polynomials, the operation  can be realized as an expected characteristic polynomial of a sum of random matrices.

##### Theorem 1.2

If $p(x)=\chi _{x} \left( A \right)$ and $q(x)=\chi _{x} \left( B \right)$ for $d\times d$ complex normal matrices *A*, *B*, then3where the expectation is over a random unitary matrix *Q* from the Haar measure on *U*(*d*).

In fact, by unitary invariance the right hand side depends only on $\chi _{x} \left( A \right)$ and $\chi _{x} \left( B \right)$ and not on the further details of *A* and *B*, so we may take them to be any normal matrices with the same characteristic polynomials.

The convolution (1) was studied by Walsh [1], who proved results including the following theorem (see also [2] and [3, Theorem 5.3.1]).

##### Theorem 1.3

If *p* and *q* are real rooted polynomials of degree *d*, then so is . Moreover,

In Theorem 1.12 we strengthen this bound on the maximum root. Our result is much tighter in the case that most of the roots of *p* and *q* are far from their maximum roots.

#### Symmetric multiplicative convolution

##### Definition 1.4

For complex univariate polynomials

$$\begin{aligned} p (x) = \sum _{i=0}^{d} x^{d-i} (-1)^{i} a_{i} \quad \text { and} \quad q (x) = \sum _{i=0}^{d} x^{d-i} (-1)^{i} b_{i} \end{aligned}$$

of degree at most *d*, the *dth symmetric multiplicative convolution* of *p* and *q* is defined as:4

It is clear that  is also bilinear, though it does not commute with differentiation or translation.

The following compact differential form of (4), analogous to (2), was discovered by B. Mirabelli [4] who has kindly allowed us to include it here: if $p(x)=P(xD)(x-1)^{d}$ and $q(x)=Q(xD)(x-1)^{d}$, then5Note that every polynomial of degree at most *d* can be written as $P(xD)(x-1)^d$, though it is not as obvious as in the additive case. The appearance of the polynomial $(x-1)^d$ is explained by the fact that it is the identity element for , i.e.,  for every *p* of degree at most *d*.

We show that for monic polynomials the operation  can be realized as an expected characteristic polynomial of a *product* of random matrices.

##### Theorem 1.5

If $p(x)=\chi _{x} \left( A \right)$ and $q(x)=\chi _{x} \left( B \right)$ for $d\times d$ complex normal matrices *A*, *B*, then6where the expectation is over a Haar unitary *Q*.

The identity element $(x-1)^d$ thereby corresponds to taking $B=I$ in the above formula.

This convolution was studied by Szegö [5], who proved the following theorem.

##### Theorem 1.6

If *p* and *q* have only nonnegative real roots, then the same is true of . Moreover,

We give a quantitatively stronger bound in Theorem 1.13.

#### Asymmetric additive convolution

##### Definition 1.7

For complex univariate polynomials

$$\begin{aligned} p (x) = \sum _{i=0}^{d} x^{d-i} (-1)^{i} a_{i}, \quad \text {and} \quad q (x) = \sum _{i=0}^{d} x^{d-i} (-1)^{i} b_{i}, \end{aligned}$$

of degree at most *d*, we define the *dth asymmetric additive convolution* of *p* and *q* to be:7

This is equivalent to the expressionwhich may also be written for $p(x)=P(DxD)x^{d}$ and $q(x)=Q(DxD)x^{d}$ asthe latter being an observation of [4].

We are not aware of previous studies of this convolution. We show that if *p*(*x*) and *q*(*x*) are monic *with all roots real and nonnegative*, then  can be realized as an expected characteristic polynomial.

##### Theorem 1.8

For two monic polynomials $p(x)=\chi _{x} \left( AA^* \right)$ and $q(x)=\chi _{x} \left( BB^* \right)$ with *A*, *B* any $d\times d$ square complex matrices,8where the expectation is taken over independent Haar unitaries *R* and *Q*.

Theorem 1.8 is a consequence of Theorem 2.14, which we prove in Sect. 2.2.2.

We prove the following real-rootedness theorem in Sect. 3.

##### Theorem 1.9

If *p* and *q* have only nonnegative real roots, then the same is true of .

We obtain bounds on the roots of  in Theorem 1.14.

##### Remark 1.10

In an earlier version of this paper [6], we *defined* the operations  and  in terms of random matrices using the formulas (3), (6), (7), and stated the formulas appearing in Definitions 1.1, 1.4, and 1.7 as theorems by showing that they were equivalent. We have chosen to reverse this presentation since  were in fact already defined by Walsh and Szegö, albeit in a different context, and their basic properties such as bilinearity are more immediate from the purely algebraic definitions.

### Motivation and related results

Before describing our analytic results on the locations of roots of the convolutions in the next section, we explain the motivations for studying them in the context of several other areas of mathematics.

*Free probability* Free probability theory (see e.g. [7–9]) studies among other things the large *d* limits of random matrices such as those in (1.1), (1.4), and (1.7). In particular, it allows one to calculate the limiting spectral distribution of a sum or product of two unitarily invariant random matrix ensembles in terms of the limiting spectral distributions of the individual ensembles. For both the sum and the product, free probability provides a transform of the moments of the spectra of the individual matrices from which one can easily derive the transform of the moments of the limiting spectra of the resulting matrices—these are known as the *R*- and *S*-transforms, which may be viewed as generating functions for certain polynomials in the moments (known as free cumulants) which are linear in the convolutions. We show in Theorems 1.12, 1.13, and 1.14 (discussed in Sect. 1.3) that for our “finite free convolutions” the same transforms provide *upper bounds* on the roots of the corresponding expected polynomials in *finite dimensions*, when evaluated at specific points on the real line.

Following the definitions in this paper, [10, 11] have shown that by taking appropriate limits our finite free convolutions yield the standard free convolutions in free probability theory. Thus, expected characteristic polynomials provide an alternative “discretization” of free convolutions from the typical one involving random matrices.

The paper [12] shows that the real zeros of  and  may be interpreted as the $\beta \rightarrow \infty$ limit of certain generalizations of $\beta -$ensembles in random matrix theory.

*Combinatorics* The original motivation for this work is the method of interlacing families of polynomials [13–15], which reduces various combinatorial problems concerning eigenvalues to problems of bounding the roots of the expected characteristic polynomials of certain random matrices. In particular, the paper [15] studies bipartite random regular graphs, whose expected characteristic polynomials turn out to be of the type appearing in (1.7). The bound of Theorem 1.14 on the roots of these polynomials then implies the existence of bipartite Ramanujan graphs of every size and every degree (a result that was later turned into a polynomial time algorithm by Cohen [16]). Hall, Puder, and Sawin [17] used some of the techniques in this paper to prove the related result that every bipartite graph has a $k-$cover which is Ramanujan, for every $k\ge 2$, generalizing [13].

#### Remark 1.11

(Renumbered Theorems) The papers [15–17] cite theorems in the original arxiv version of this work [6], which are numbered differently from the ones in this paper. In particular, Theorems 1.7 and 1.8 of [6] correspond to Theorems 1.12 and 1.14 of this paper.

*Representation theory* As shown in Sect. 2, the unitary group may be replaced by the orthogonal group or the group of signed permutations in Theorems 1.2, 1.5, and 1.8 without changing the expected characteristic polynomial and therefore any of their statements. The ability to compute the average of a matrix function over the group of unitary matrices by instead computing an average over some smaller group of matrices is a phenomenon we refer to as *quadrature* (see [15] for more details).

The proofs given Sect. 2 are different from those that appeared in the first version [6] of this paper. The original proofs treat each of the convolutions as (specifically) being an average over the unitary group, and then by explicit calculations show that one can (in some cases) replace the unitary group with the signed permutation matrices and get the same result. After posting that preprint, we were informed [17] that our quadrature results were actually a manifestation of well-studied concepts in representation theory (the Peter-Weyl theorem); the work [17] gave a more general sufficient condition for a subgroup of the unitary group to have this property .

One subgroup that has been used specifically in an application is the $n\times n$ matrices corresponding to the standard representation of $S_{n+1}$ (the symmetric group on $n+1$ elements). The fact that this group is a valid quadrature group plays a pivotal role in the results on Ramanujan graphs [15] and [17] mentioned above.

*Geometry of polynomials* Theorem 1.3 implies that if *p*(*x*) is real-rooted of degree *d*, then the linear transformation  preserves real-rootedness of all real polynomials of degree at most *d*. Leake and Ryder [18] observe that a partial converse is also true: every differential operator $T:{\mathbb {R}}_{\le d}[x]\rightarrow {\mathbb {R}}_{\le d}[x]$ preserving real-rootedness can be written as  for some *p* of degree at most *d*. Thus, our bounds on the extreme roots of the additive convolution imply bounds on how much any such operator can perturb the roots of its real rooted inputs. They also generalize our analytic bounds on the roots of symmetric additive convolutions (Theorem 1.12) by showing that they are a special case of submodularity relation.

*Random matrix theory* The expected characteristic polynomials of symmetric Gaussian random matrices are Hermite polynomials (see, e.g. [19, Theorem 4.1]). If *R* is an *d*-by-*d* matrix of independent Gaussian random variables of variance 1, then

$$\begin{aligned} {\mathbb {E}}\chi _{x} \left( \frac{R + R^T}{\sqrt{2}} \right) = H_d (x), \end{aligned}$$

where

$$\begin{aligned} H_d (x) = e^{-D^2 / 2} x^d \end{aligned}$$

is the *d*th Hermite polynomial. By applying Theorem 1.2, but taking the expectation over orthogonal matrices, or by applying the formula (2), we may conclude that for positive *a* and *b* and $c = \sqrt{a^2 + b^2}$,Similarly, the expected characteristic polynomial of $R R^T$ is the *d*th Laguerre polynomial [20, Sect. 9]

$$\begin{aligned} L_d (x) = \left( 1 - D \right) ^d x^d. \end{aligned}$$

Thus, both Theorem 1.8 and the definition (7) can be used to show that for postive *a* and *b* and $c = a + b$,

### Transforms and root bounds

In free probability, each of the three convolutions comes equipped with a natural transform of probability measures. We define analogous transforms on polynomials and use them to bound the extreme roots of the convolutions of polynomials.

We will identify a vector $(\lambda _{1}, \dotsc , \lambda _{d})$ with the discrete distribution that takes each value $\lambda _{i}$ with probability 1/*d*. The Cauchy/Hilbert/Stieltjes transform of such a distribution is the function

$$\begin{aligned} {\mathcal {G}}_{\lambda } \left( x \right) = \frac{1}{d} \sum _{i=1}^{d} \frac{1}{x-\lambda _{i}}. \end{aligned}$$

Given a polynomial *p* with roots $\lambda _{1}, \dotsc , \lambda _{d}$, we similarly define

$$\begin{aligned} {\mathcal {G}}_{p} \left( x \right) := {\mathcal {G}}_{\lambda } \left( x \right) . \end{aligned}$$

We will prove theorems about the inverse of the Cauchy transform, which we define for real $w>0$ by

$$\begin{aligned} {\mathcal {K}}_{p} \left( w \right) := \max \left\{ x : {\mathcal {G}}_{p} \left( x \right) = w \right\} . \end{aligned}$$

For a real rooted polynomial *p*, and thus for real $\lambda _{1}, \dotsc , \lambda _{d}$, this is the value of *x* that is larger than all the $\lambda _{i}$ for which ${\mathcal {G}}_{p} \left( x \right) = w$. Since ${\mathcal {G}}_{p} \left( x \right)$ is decreasing above the largest root of *p*, the maximum is uniquely attained for each $w>0$.

As $${\mathcal {G}}_{p} \left( x \right) = \frac{1}{d} \frac{p' (x)}{p (x)}$$,

$$\begin{aligned} {\mathcal {G}}_{p} \left( x \right) = w \quad \iff \quad p (x) - \frac{1}{w d} p' (x) = 0. \end{aligned}$$

This tells us that

$$\begin{aligned} {\mathcal {K}}_{p} \left( w \right) = \mathsf {maxroot} \left( U_{1/wd} p \right) , \end{aligned}$$

where we define

$$\begin{aligned} U_{\alpha } p (x) := p (x) - \alpha Dp (x). \end{aligned}$$

Voiculescu’s R-transform of the probability distribution that is uniform on $\lambda$ is given by ${\mathcal {R}}_{\lambda } \left( w \right) = {\mathcal {K}}_{\lambda } \left( w \right) - 1/w$ (though in free probability the inversion is typically done at the level of power series). We use the same notation to define a transform on polynomials

$$\begin{aligned} {\mathcal {R}}_{p} \left( w \right) := {\mathcal {K}}_{p} \left( w \right) - 1/w. \end{aligned}$$

If $\lambda$ and $\mu$ are compactly supported probability distributions on the reals, then their free convolution  satisfies [7]:For our finite additive convolution, we obtain an analogous *inequality* for $w>0$.

#### Theorem 1.12

For $w > 0$ and real-rooted polynomials *p* and *q* of degree *d*,9with equality if and only if *p*(*x*) or *q*(*x*) has the form $(x-\lambda )^{d}$.

We will often write (9) as:where $\alpha = 1/wd$.

To bound the roots of the finite multiplicative convolution, we employ a variant of Voiculescu’s $S-$transform. We first define a variant of the moment transform, which we write as a power series in 1/*z* instead of in *z*:

$$\begin{aligned} \widetilde{{\mathcal {M}}}_{p} \left( z \right) := z {\mathcal {G}}_{p} \left( z \right) - 1 = \frac{1}{d} \sum _{i=1}^{d} \sum _{j \ge 1} \left( \frac{\lambda _{i}}{z} \right) ^{j}. \end{aligned}$$

For a polynomial *p* having only nonnegative real roots and a $z > 0$,

$$\begin{aligned} z > \mathsf {maxroot} \left( p \right) \quad \iff \quad \widetilde{{\mathcal {M}}}_{p} \left( z \right) < \infty . \end{aligned}$$

We define the inverse of this transform, $\widetilde{{\mathcal {M}}}^{(-1)}_{p} \left( w \right)$, to be the largest *z* so that $\widetilde{{\mathcal {M}}}_{p} \left( z \right) = w$, and

$$\begin{aligned} \widetilde{{\mathcal {S}}}_{p} \left( w \right) = \frac{w}{w+1} \widetilde{{\mathcal {M}}}^{(-1)}_{p} \left( w \right) . \end{aligned}$$

This is the reciprocal of the usual *S*-transform. We prove the following bound on this transformation in Sect. 4.2.

#### Theorem 1.13

For degree *d* polynomials *p* and *q* having only nonnegative real roots and $w > 0$,with equality only when *p* or *q* has only one distinct root.

One can ask whether an inequality similar to Theorem 1.13 could hold in more generality (i.e., for a larger collection of polynomials than just those with nonnegative roots). While this seems possible, in this paper we will restrict to the case that *both* polynomials have nonnegative roots (see Remark 4.10 in Sect. 4.2).

To define the relevant transform for the asymmetric additive convolution, we define ${\mathbb {S}}$ to be the linear map taking a polynomial *p*(*x*) to $p (x^{2})$. If *p* has only positive real roots $\lambda _{i}$, then ${\mathbb {S}}p$ has roots $\pm \sqrt{\lambda _{i}}$. If $\lambda$ is a probability distribution supported on the nonnegative reals, then we use ${\mathbb {S}}\lambda$ to denote the corresponding symmetric probability distribution on $\pm \sqrt{\lambda _{i}}$. If $\lambda$ and $\mu$ are compactly supported probability distributions on the positive reals, then Benaych-Georges [21] showed that their appropriately defined rectangular convolution  satisfies:In Sect. 4.3 we derive an analog of this result in the form of the following inequality.

#### Theorem 1.14

For degree *d* polynomials *p* and *q* having only nonnegative real roots and $w>0$,

#### Remark 1.15

The formulas above are stated only for polyomials of degree exactly *d*, but they may be applied to polynomials of degree at most *d* by first applying the degree-reduction formulas outlined in the next section.

### Polynomials of different degrees

The operation  is defined above for pairs of polynomials *p* and *q* of degree at most *d*, but if one or both of the polynomials has degree $c<d$, it may be written in terms of the lower degree operation .

#### Lemma 1.16

(Degree Reduction for ) Suppose $\deg (p)\le d$ and $\deg (q)\le d-1$. Then:10

#### Proof

By (2), the differential operator  is equal to ${\hat{p}}(D)$ where ${\hat{p}}(D)x^d=p(x)$. Differentiating, we have

$$\begin{aligned} {\hat{p}}(D)x^{d-1}=(D/d){\hat{p}}(D)x^d = (Dp/d)(x), \end{aligned}$$

so we must have  whenever $\deg (q)\le d-1$. $\square$

This relationship between convolutions of different grades turns out to be a key tool in the analytic proofs in Sect. 4, which induct on the degrees of the polynomials. We prove similar degree reduction formulas for  and  in Lemmas 4.9 and 4.16, but have chosen to present them in context along with the inductive proofs in Sect. 4.

It turns out that the operation  can be naturally defined for polynomials of degree strictly greater than *d* via the random matrix identity (3). The key observation is that the expected characteristic polynomial of a random restriction of a $d\times d$ matrix is proportional to a derivative of its characteristic polynomial.

#### Lemma 1.17

If $a > d$, *A* is an $a\times a$ matrix and *Q* is a random $d\times a$ complex matrix with orthonormal rows (i.e., sampled from the Haar measure on the complex $d\times a$ Stiefel manifold ${\mathbb {C}}^{d}_{a}$), then

$$\begin{aligned} {\mathbb {E}}_{Q} \chi _{x} \left( Q A Q^{*} \right) = \frac{d!}{a!}D^{a-d}q(x). \end{aligned}$$

We prove this lemma in Sect. 2. Lemma 1.17 yields the following corollary, which may be viewed as a generalization of the definition of  to polynomials of degree greater than *d*.

#### Corollary 1.18

If *A* and *B* are $a\times a$ and $b\times b$ Hermitian matrices with $a,b\ge d$ and characteristic polynomials *p*(*x*) and *q*(*x*) respectively, and *R*, *Q* are uniformly random from ${\mathbb {C}}^{d}_{a}$ and ${\mathbb {C}}^{d}_{b}$ respectively, then11

#### Proof

Since the formula (1) is bilinear in the characteristic polynomials $\chi _{x} \left( A \right)$ and $\chi _{x} \left( B \right)$, we have for fixed *R*:Averaging over *R* and invoking bilinearity and Lemma 1.17 once more finishes the proof. $\square$

Note that the definition (11) is consistent with Lemma 1.16, e.g. if $b=d$ then the right hand side of (11) is equal to . As differentiation preserves real-rootedness, the generalized definition of  preserves real-rootedness of polynomials of all degrees, and bounds on their roots may be obtained from our results by reducing to the equal degree case by differentiating sufficiently many times.

While Lemma 1.17 can be used in conjunction with  and  just as easily due to their bilinearity, this does not correspond to the appropriate degree-reduction operators (see Lemmas 4.9 and 4.16) for those cases, so it does not lead to a satisfying generalization of the definitions to higher degrees.

### Notation and organization

Let ${\mathbb {P}} (d)$ be the family of real rooted polynomials of degree exactly *d* with positive leading coefficient, and let ${\mathbb {P}}$ be the union over *d* of ${\mathbb {P}} (d)$. Let ${\mathbb {P}}^{+} (d)$ be the subset of these polynomials having only nonnegative roots. We let ${\mathbb {P}}^{+}$ be the union of ${\mathbb {P}}^{+} (d)$ over all $d \ge 1$.

For a function *f*(*x*), we write the derivative as $Df (x)$. For a number $\alpha$, we let $U_{\alpha }$ be the operator that maps *f* to $f - \alpha D f$. That is, $U_{\alpha }$ is multiplication by $1 - \alpha D$.

## Equivalence of convolutions and ${\mathbb {E}}\chi$

The goal of this section is to prove Theorems 1.2, 1.5, and 1.8 relating the three convolutions to random matrices. While we have so far only considered averages over unitary matrices, it turns out that one can average over various other collections of matrices and get the same formula. In Sect. 2.1, we will define a property that we call *minor-orthogonality* and then in Sect. 2.2 we will show that the coefficients we are interested can be computed using an average over any collection of minor-orthogonal matrices. Also in Sect. 2.1, we will show that the collection of $n \times n$ signed permutation matrices (under a uniform distribution) is minor-orthogonal, and then we will use this to show that the orthogonal matrices (under the Haar measure) is minor-orthogonal.

There are some advantages to being able to express the convolutions as averages over different collections of matrices; in particular, a formula that is easily derived by replacing a unitary average by one over signed permutation matrices will be used in the proof of Lemma 4.25.

### Minor-orthogonality

We will write [*n*] to denote the set $\{ 1, \dots , n \}$ and for a set *S*, we write $$\left( {\begin{array}{c}S\\ k\end{array}}\right) $$ to denote the collection of subsets of *S* that have exactly *k* elements. When our sets contain integers (which they always will), we will consider the set to be ordered from smallest to largest. Hence, for example, if *S* contains the elements $\{ 2, 5, 3 \}$, then we will write

$$\begin{aligned} S = \{ s_1, s_2, s_3 \} \quad \text {where} \quad s_1 = 2, s_2 = 3, s_3 = 5. \end{aligned}$$

Now let $$S = \{ s_1, \dots , s_k \} \in \left( {\begin{array}{c}[n]\\ k\end{array}}\right) $$. For a set $$W \in \left( {\begin{array}{c}[k]\\ j\end{array}}\right) $$ with $j \le k$, we will write

$$\begin{aligned} W(S) = \{ s_i : i \in W \}. \end{aligned}$$

Lastly, for a set of integers *S*, we will write

$$\begin{aligned} \Vert S \Vert _1 = \sum _{s \in S} s. \end{aligned}$$

#### Example 2.1

For $W = \{ 1, 3 \}$ and $S = \{ 2, 4, 5 \}$ we have

$$\begin{aligned} W(S) = \{ 2, 5 \} \wedge \Vert W\Vert _1 = 1 + 3 = 4 \wedge \Vert S\Vert _1 = 2 + 4 + 5 = 11. \end{aligned}$$

Let $m, n$ be positive integers. Given an $m\times n$ matrix *A* and sets $S \subseteq [m], T \subseteq [n]$ with $|S | = |T |$, we will write the *(S, T)-minor of A* as

$$\begin{aligned}{}[A]_{S, T} = \det \left( \{ a_{i, j} \}_{i \in S, j \in T}\right) \end{aligned}$$

By definition, we will set $[A]_{\varnothing , \varnothing } = 1$. A well-known theorem of Cauchy and Binet relates the minor of a product to the product of minors ( [22]):

#### Theorem 2.2

For integers *m*, *n*, *p*, *k* and $m \times n$ matrix *A* and $n \times p$ matrix *B*, we have

12 $$\begin{aligned}{}[AB]_{S, T} = \sum _{|U | \in \left( {\begin{array}{c}[n]\\ k\end{array}}\right) } [A]_{S, U} [B]_{U, T} \end{aligned}$$

for any sets $$S \in \left( {\begin{array}{c}[m]\\ k\end{array}}\right) $$ and $$T \in \left( {\begin{array}{c}[p]\\ k\end{array}}\right) $$.

#### Definition 2.3

We will say that an $m\times n$ random matrix *R* is *minor-orthogonal* if for all integers $k, \ell \le \max \{ m, n\}$ and all sets *S*, *T*, *U*, *V* with $|S | = |T | = k$ and $|U | = |V | = \ell$, we have

$$\begin{aligned} {\mathbb {E}}_{R} \left\{ \, [R]_{S, T}[R^*]_{U, V} \,\right\} = \frac{1}{\left( {\begin{array}{c} \max \{ m, n\} \\ k\end{array}}\right) }\delta _{ \{ S = V \} } \delta _{ \{ T = U \} } . \end{aligned}$$

Given a minor-orthogonal matrix *R* it is easy to see from the definition that

1. $R^*$ is minor orthogonal
2. any submatrix that preserves the largest dimension of *R* is minor orthogonal

#### Lemma 2.4

If *R* is minor-orthogonal and *Q* is a fixed matrix for which $QQ^* = I$, then *QR* is minor-orthogonal. If $Q^*Q=I$ then *RQ* is minor orthogonal.

#### Proof

For any sets *S*, *T* with , we have

$$\begin{aligned}{}[QR]_{S, T} = \sum _{|W | = k} [Q]_{S, W} [R]_{W, T} \end{aligned}$$

so for $|U | = |V | = \ell$, we have

$$\begin{aligned} {\mathbb {E}}_{R} \left\{ \, [QR]_{S, T}[(QR)^*]_{U, V} \,\right\}&= {\mathbb {E}}_{R} \left\{ \, \sum _{|W | = k}\sum _{|Z | = \ell } [Q]_{S, W} [R]_{W, T} [R^*]_{U, Z} [Q^*]_{Z, V} \,\right\} \\ {}&= \sum _{|W | = k}\sum _{|Z | = \ell } [Q]_{S, W} [Q^*]_{Z, V} \frac{1}{\left( {\begin{array}{c} \max \{ m, n\} \\ k\end{array}}\right) }\delta _{ \{ W = Z \} } \delta _{ \{ T = U \} } \\ {}&= \sum _{|W | = k} \frac{1}{\left( {\begin{array}{c} \max \{ m, n\} \\ k\end{array}}\right) }[Q]_{S, W} [Q^*]_{W, V} \delta _{ \{ T = U \} } \\ {}&= \frac{1}{\left( {\begin{array}{c} \max \{ m, n\} \\ k\end{array}}\right) }\delta _{ \{ S = V \} } \delta _{ \{ T = U \} } , \end{aligned}$$

where the last line comes from the fact that $[I]_{S, V} = \delta _{ \{ S = V \} }$.

The other case *RQ* follows by repeating the argument with $R^*$ and noting that $RQ=(Q^*R^*)^*$. $\square$

#### Definition 2.5

A *signed permutation matrix* is a matrix that can be written *EP* where *E* is a diagonal matrix with $\pm 1$ diagonal entries and *P* is a permutation matrix.

#### Lemma 2.6

A uniformly random $n\times n$ signed permutation matrix is minor-orthogonal.

#### Proof

We can write a uniformly random signed permutation matrix *Q* as $Q = E_\chi P_\pi$ where $P_\pi$ is a uniformly random permutation matrix and  is a uniformly random diagonal matrix with $\chi \in \{ \pm 1 \}^n$ on the diagonal (and the two are independent). Hence for $|S | = |T | = k$ and $|U | = |V | = \ell$, we have

$$\begin{aligned} {\mathbb {E}}_{Q} \left\{ \, [Q]_{S, T}[Q^*]_{U, V} \,\right\}&= {\mathbb {E}}_{\chi , \pi } \left\{ \, [E_\chi P_\pi ]_{S, T} [ P_\pi ^* E_\chi ]_{U, V} \,\right\} \\ {}&= \sum _{|W | = k}\sum _{|Z | = \ell } {\mathbb {E}}_{\chi , \pi } \left\{ \, [E_\chi ]_{S, W} [P_\pi ]_{W, T} [ P_\pi ^* ]_{U, Z} [E_\chi ]_{Z, V} \,\right\} . \\ {}&= {\mathbb {E}}_{\chi , \pi } \left\{ \, [E_\chi ]_{S, S} [P_\pi ]_{S, T} [ P_\pi ^* ]_{U, V} [E_\chi ]_{V, V} \,\right\} \\ {}&{\mathbb {E}}_{\chi } \left\{ \, \prod _{i \in S} \chi _i \prod _{j \in V} \chi _j \,\right\} {\mathbb {E}}_{\pi } \left\{ \, [P_\pi ]_{S, T} [ P_\pi ^* ]_{U, V} \,\right\} . \end{aligned}$$

where the penultimate line uses the fact that a diagonal matrix *X* satisfies $[X]_{A, B} = 0$ whenever . Now the $\chi _i$ are uniformly distributed $\{ \pm 1 \}$ random variables, so

$$\begin{aligned} {\mathbb {E}}_{\chi } \left\{ \, \prod _{i \in S} \chi _i \prod _{j \in V} \chi _j \,\right\} = \delta _{ \{ S = V \} } \end{aligned}$$

and so we have

$$\begin{aligned} {\mathbb {E}}_{Q} \left\{ \, [Q]_{S, T}[Q^*]_{U, V} \,\right\}&= {\mathbb {E}}_{\pi } \left\{ \, [P_\pi ]_{S, T} [ P_\pi ^* ]_{U, V} \,\right\} \delta _{ \{ S = V \} } \\ {}&= {\mathbb {E}}_{\pi } \left\{ \, [P_\pi ]_{S, T} [ P_\pi ]_{S, U} \,\right\} \delta _{ \{ S = V \} } \end{aligned}$$

Furthermore, $[P_\pi ]_{S, T} = 0$ except when $T = \pi (S)$, so in order for both $[P_\pi ]_{S, T}$ and $[ P_\pi ]_{S, U}$ to be nonzero simultaneously requires $U = T$. In the case that $U = T = \pi (S)$, $[P_\pi ]_{S, T} = \pm 1$, and so we have

$$\begin{aligned} {\mathbb {E}}_{Q} \left\{ \, [Q]_{S, T}[Q^*]_{U, V} \,\right\}&= {\mathbb {E}}_{\pi } \left\{ \, [P_\pi ]_{S, T}^2 \,\right\} \delta _{ \{ S = V \} } \delta _{ \{ T = U \} } \\ {}&= {\mathbb {E}}_{\pi } \left\{ \, \delta _{ \{ \pi (S) = T \} } \,\right\} \delta _{ \{ S = V \} } \delta _{ \{ T = U \} } \end{aligned}$$

The probability that a permutation of length $n$ maps a set *S* to a set *T* with $|S | = |T | = k$ is

$$\begin{aligned} \frac{k!(n-k)!}{n!} = \frac{1}{\left( {\begin{array}{c}n\\ k\end{array}}\right) } \end{aligned}$$

and so for $|S | = |T | = k$, we have

$$\begin{aligned} {\mathbb {E}}_{Q} \left\{ \, [Q]_{S, T}[Q^*]_{U, V} \,\right\} = \frac{1}{\left( {\begin{array}{c}n\\ k\end{array}}\right) } \delta _{ \{ S = V \} } \delta _{ \{ T = U \} } \end{aligned}$$

as required. The case $m> n$ follows by considering $R^*$ instead of *R*. $\square$

#### Corollary 2.7

An $m\times n$ Haar random matrix from the Stiefel manifold ${\mathbb {C}}^{m}_{n}$ is minor-orthogonal.

#### Proof

Let *R* be such a random matrix, and assume $m\le n$. As a signed permutation matrix is unitary, *RQ* is also Haar distributed for any fixed signed permutation matrix *Q*. By Lemma 2.4 it is also minor-orthogonal. Hence

$$\begin{aligned} {\mathbb {E}}_{R} \left\{ \, [R]_{S, T}[R^*]_{U, V} \,\right\} = {\mathbb {E}}_{R} \left\{ \, [R Q]_{S, T}[(RQ)^*]_{U, V} \,\right\} \end{aligned}$$

and so

$$\begin{aligned} {\mathbb {E}}_{R} \left\{ \, [R]_{S, T}[R^*]_{U, V} \,\right\} = {\mathbb {E}}_{R, Q} \left\{ \, [R]_{S, T}[R^*]_{U, V} \,\right\} = {\mathbb {E}}_{R, Q} \left\{ \, [RQ]_{S, T}[(RQ)^*]_{U, V} \,\right\} , \end{aligned}$$

where *Q* is a uniform signed permutation independent from *R*. By Lemma 2.6, *Q* is minor-orthogonal and so, for fixed *R*, Lemma 2.4 implies that *RQ* is minor-orthogonal. So

$$\begin{aligned} {\mathbb {E}}_{R} \left\{ \, {\mathbb {E}}_{Q} \left\{ \, [RQ]_{S, T}[(RQ)^*]_{U, V} \,\right\} \,\right\} = {\mathbb {E}}_{R} \left\{ \, \frac{1}{\left( {\begin{array}{c}n\\ k\end{array}}\right) } \delta _{ \{ S = V \} } \delta _{ \{ T = U \} } \,\right\} = \frac{1}{\left( {\begin{array}{c}n\\ k\end{array}}\right) } \delta _{ \{ S = V \} } \delta _{ \{ T = U \} } \end{aligned}$$

as required. $$\square $$

### Formulas

We begin this section by mentioning a well-known formula for the determinants of a sum of matrices (see [23]):

#### Theorem 2.8

For integers $k \le n$, $n\times n$ matrices *A*, *B*, and sets $$S, T \in \left( {\begin{array}{c}[n]\\ k\end{array}}\right) $$, we have

13 $$\begin{aligned}{}[A + B]_{S, T} = \sum _{i=0}^{k} \sum _{U, V \in \left( {\begin{array}{c}[k]\\ i\end{array}}\right) } (-1)^{\Vert U\Vert _1 + \Vert V \Vert _1} [A]_{U(S), V(T)} [B]_{\overline{U}(S), \overline{V}(T)}, \end{aligned}$$

where $$\overline{U} = [k] \setminus U$$.

We denote the coefficient of $(-1)^{k} x^{d-k}$ of the characteristic polynomial of a *d*-dimensional matrix *A* by $\sigma _{k} (A)$, which we recall is the *k*th elementary symmetric function of the eigenvalues of *A*. We will repeatedly use the fact that

14 $$\begin{aligned} \sigma _{k}(A) = \sum _{|S |=k}[A]_{S, S}. \end{aligned}$$

#### Lemma 2.9

For integers $m \le n$, let *B* be an $n \times n$ matrix and let *R* be a $m \times n$ minor-orthogonal matrix independent from *B*. Then for all $|S | = |T | = k$, we have

$$\begin{aligned} {\mathbb {E}}_{R} \left\{ \, [R B R^*]_{S, T} \,\right\} = \delta _{ \{ S = T \} } \frac{\sigma _k(B)}{\left( {\begin{array}{c}n\\ k\end{array}}\right) } \end{aligned}$$

#### Proof

By Theorem 2.2,

$$\begin{aligned} {\mathbb {E}}_{R} \left\{ \, [R B R^*]_{S, T} \,\right\}&= \sum _{X, Y \in \left( {\begin{array}{c}[n]\\ k\end{array}}\right) } {\mathbb {E}}_{R} \left\{ \, [R]_{S, X} [B]_{X, Y} [R^*]_{Y, T} \,\right\} \\ {}&= \sum _{X, Y \in \left( {\begin{array}{c}[n]\\ k\end{array}}\right) } [B]_{X, Y} {\mathbb {E}}_{R} \left\{ \, [R]_{S, X} [R]_{T, Y} \,\right\} \\ {}&= \sum _{X, Y \in \left( {\begin{array}{c}[n]\\ k\end{array}}\right) } [B]_{X, Y} \frac{1}{\left( {\begin{array}{c}n\\ k\end{array}}\right) } \delta _{ \{ X = Y \} } \delta _{ \{ S = T \} } \\ {}&= \sum _{X \in \left( {\begin{array}{c}[n]\\ k\end{array}}\right) } [B]_{X, X} \frac{1}{\left( {\begin{array}{c}n\\ k\end{array}}\right) } \delta _{ \{ S = T \} } . \end{aligned}$$

$$\square $$

The above lemma yields a quick proof of Lemma 1.17.

#### Proof of Lemma 1.17

Let *A* be an $a\times a$ matrix and let *Q* be Haar distributed on ${\mathbb {C}}^{d}_{a}$. The $k^{th}$ coefficient of ${\mathbb {E}}\chi _{x} \left( QAQ^* \right)$ is equal to

$$\begin{aligned} {\mathbb {E}}_{Q} \left\{ \, \sigma _k(QAQ^*) \,\right\}&= \sum _{|S |=k} {\mathbb {E}}_{Q} \left\{ \, [QAQ^*]_{S,S} \,\right\} \\&= \sum _{|S |=k}\frac{\sigma _k(A)}{\left( {\begin{array}{c}a\\ k\end{array}}\right) }\quad \hbox { by Lemma 2.9 and minor-orthogonality of}\ Q\\&= \frac{\left( {\begin{array}{c}d\\ k\end{array}}\right) }{\left( {\begin{array}{c}a\\ k\end{array}}\right) }, \end{aligned}$$

which is precisely the $k^{th}$ coefficient of $\frac{d!}{a!}D^{a-d}\chi _{x} \left( Q \right)$. $\square$

#### Symmetric additive and multiplicative convolutions

Using Lemma 2.9, we can easily prove Theorems 1.2 and 1.5 by showing equality of each coefficient as per (14).

##### Theorem 2.10

(Implies Theorem 1.2) Let *A* and *B* be $d\times d$ matrices, and let *R* be a random $d\times d$ minor-orthogonal matrix. Then

$$\begin{aligned} {\mathbb {E}}_{R} \left\{ \, \sigma _k(A + R B R^*) \,\right\} = \sum _{i=0}^k \frac{\left( {\begin{array}{c}d-i\\ k-i\end{array}}\right) }{\left( {\begin{array}{c}d\\ k-i\end{array}}\right) }\sigma _i(A)\sigma _{k-i}(B). \end{aligned}$$

##### Proof

By (14), Theorem 2.8, and then Lemma 2.9, we have

$$\begin{aligned}&{\mathbb {E}}_{R} \left\{ \, \sigma _k(A + R B R^*) \,\right\} \\&\quad = \sum _{S \in \left( {\begin{array}{c}[d]\\ k\end{array}}\right) } {\mathbb {E}}_{R} \left\{ \, [A + R B R^*]_{S, S} \,\right\} \\&\quad = \sum _{S \in \left( {\begin{array}{c}[d]\\ k\end{array}}\right) } \sum _{i=0}^{k} \sum _{U, V \in \left( {\begin{array}{c}[k]\\ i\end{array}}\right) } (-1)^{\Vert U\Vert _1 + \Vert V \Vert _1} [A]_{U(S), V(S)} {\mathbb {E}}_{R} \left\{ \, [R B R^*]_{\overline{U}(S), \overline{V}(S)} \,\right\} \\&\quad = \sum _{S \in \left( {\begin{array}{c}[d]\\ k\end{array}}\right) } \sum _{i=0}^{k} \sum _{U, V \in \left( {\begin{array}{c}[k]\\ i\end{array}}\right) } (-1)^{\Vert U \Vert _1 + \Vert V \Vert _1} [A]_{U(S), V(S)} \delta _{ \{ \overline{U}(S) = \overline{V}(S) \} } \frac{\sigma _{k-i}(B)}{\left( {\begin{array}{c}d\\ k-i\end{array}}\right) } \\&\quad = \sum _{i=0}^{k} \frac{\sigma _{k-i}(B)}{\left( {\begin{array}{c}d\\ k-i\end{array}}\right) } \sum _{S \in \left( {\begin{array}{c}[d]\\ k\end{array}}\right) } \sum _{U \in \left( {\begin{array}{c}[k]\\ i\end{array}}\right) } [A]_{U(S), U(S)} \end{aligned}$$

where the last equality uses the fact that $\overline{U}(S) = \overline{V}(S)$ if and only if $U = V$. Finally,

$$\begin{aligned} \sum _{S \in \left( {\begin{array}{c}[d]\\ k\end{array}}\right) } \sum _{U \in \left( {\begin{array}{c}[k]\\ i\end{array}}\right) } [A]_{U(S), U(S)} = \left( {\begin{array}{c}d-i\\ k-i\end{array}}\right) \sum _{V \in \left( {\begin{array}{c}[d]\\ i\end{array}}\right) } [A]_{V, V} \end{aligned}$$

as $$\left( {\begin{array}{c}d-i\\ k-i\end{array}}\right) $$ is the number of times a set *V* appears as $V = U(S)$ for some *S* and some *U*. That is, the number of ways we can add elements to a set of size *V* to obtain a set of size *k*. $\square$

Using Theorem 2.10 and Lemma 2.6 we can derive another useful formula for the symmetric additive convolution, this time as a function of the *roots*. It states that  is the average of all polynomials you can form by adding the roots of *p* and *q* pairwise.

##### Theorem 2.11

For $p(x) = \prod _{i=0}^d (x - a_i)$ and $q(x) = \prod _{i=0}^d (x - b_i)$ we havewhere the sum is over permutations $\sigma$ of [*d*].

##### Proof

Let *A* be the diagonal matrix with diagonal elements $\{ a_i \}$ and let *B* be the diagonal matrix with diagonal elements $\{ b_i \}$. By Theorem 2.10, we havewhere the expectation can be taken over any minor-orthogonal random matrix *R*. By Lemma 2.6, we can take *R* to be a (uniformly random) signed permutation matrix. Since *A* and *B* are diagonal, it is easy to compute (for each fixed value of *R*)

$$\begin{aligned} \det \left( xI - A - R B R^*\right) = \prod _i (x - a_i - b_{\sigma (i)}) \end{aligned}$$

where $\sigma$ is the permutation part of *R* (all of the signs cancel). Averaging over these gives the result. $\square$

##### Theorem 2.12

(Implies Theorem 1.5) Let *A* and *B* be $d\times d$ matrices, and let *R* be an $d\times d$ minor-orthogonal matrix. Then

$$\begin{aligned} {\mathbb {E}}_{R} \left\{ \, \sigma _k(A R B R^*) \,\right\} = \frac{\sigma _k(A)\sigma _k(B)}{\left( {\begin{array}{c}d\\ k\end{array}}\right) }. \end{aligned}$$

##### Proof

By Theorem 2.2 and then Lemma 2.9, we have

$$\begin{aligned} {\mathbb {E}}_{R} \left\{ \, \sigma _k(A R B R^*) \,\right\}&= \sum _{S \in \left( {\begin{array}{c}[d]\\ k\end{array}}\right) } {\mathbb {E}}_{R} \left\{ \, [A R B R^*]_{S, S} \,\right\} \\&= \sum _{S, T \in \left( {\begin{array}{c}[d]\\ k\end{array}}\right) } [A]_{S, T} {\mathbb {E}}_{R} \left\{ \, [R B R^*]_{T, S} \,\right\} \\&= \sum _{S, T \in \left( {\begin{array}{c}[d]\\ k\end{array}}\right) } [A]_{S, T} \delta _{ \{ T = S \} } \frac{\sigma _k(B)}{\left( {\begin{array}{c}d\\ k\end{array}}\right) } \\&= \frac{\sigma _k(A)\sigma _k(B)}{\left( {\begin{array}{c}d\\ k\end{array}}\right) }. \end{aligned}$$

$$\square $$

#### Asymmetric additive convolution

The proof of the asymmetric convolution is a bit more involved, due to the appearance of a second random matrix.

##### Lemma 2.13

Let *B* be a $d\times d$ matrix and let *Q* and *R* be $d\times d$ independently random minor-orthogonal matrices. Then

$$\begin{aligned} {\mathbb {E}}_{Q, R} \left\{ \, [Q B R^*]_{S, T} [(Q B R^*)^*]_{U, V} \,\right\} = \frac{ \delta _{ \{ T = U \} } \delta _{ \{ S = V \} } }{\left( {\begin{array}{c}d\\ k\end{array}}\right) \left( {\begin{array}{c}d\\ k\end{array}}\right) } \sigma _k(BB^*) \end{aligned}$$

for any $|S | = |T | = k$ and $|U | = |V | = \ell$.

##### Proof

By Theorem 2.2 we have

$$\begin{aligned} [Q B R^*]_{S, T} [(Q B R^*)^*]_{U, V} \\ = \sum _{|W |=|Z | = k} \sum _{|W' |=|Z' | = \ell } [Q]_{S, W}[B]_{W, Z}[R^*]_{Z, T}[R]_{U, W'}[B^*]_{W', Z'}[Q^*]_{Z', V} \end{aligned}$$

where

$$\begin{aligned} {\mathbb {E}}_{Q} \left\{ \, [Q]_{S, W}[Q^*]_{Z', V} \,\right\} = \frac{1}{\left( {\begin{array}{c}d\\ k\end{array}}\right) }\delta _{ \{ S = V \} } \delta _{ \{ W = Z' \} } \end{aligned}$$

and

$$\begin{aligned} {\mathbb {E}}_{R} \left\{ \, [R^*]_{Z, T}[R]_{U, W'} \,\right\} = \frac{1}{\left( {\begin{array}{c}d\\ \ell \end{array}}\right) }\delta _{ \{ T = U \} } \delta _{ \{ Z = W' \} } . \end{aligned}$$

Hence

$$\begin{aligned} {\mathbb {E}}_{Q, R} \left\{ \, [Q B R^*]_{S, T} [(Q B R^*)^*]_{U, V} \,\right\}&= \sum _{|W |=|Z |= k} \frac{1}{\left( {\begin{array}{c}d\\ k\end{array}}\right) } \frac{1}{\left( {\begin{array}{c}d\\ k\end{array}}\right) } [B]_{W, Z}[B^*]_{Z, W}\delta _{ \{ T = U \} } \delta _{ \{ S = V \} } \\ {}&= \sum _{|W |=k} \frac{1}{\left( {\begin{array}{c}d\\ k\end{array}}\right) } \frac{1}{\left( {\begin{array}{c}d\\ k\end{array}}\right) } [BB^*]_{W, W} \delta _{ \{ T = U \} } \delta _{ \{ S = V \} } \\ {}&= \frac{1}{\left( {\begin{array}{c}d\\ k\end{array}}\right) } \frac{1}{\left( {\begin{array}{c}d\\ k\end{array}}\right) } \sigma _k(BB^*) \delta _{ \{ T = U \} } \delta _{ \{ S = V \} } \end{aligned}$$

$$\square $$

##### Theorem 2.14

(Implies Theorem 1.8) Let *A* and *B* be $\AA \times d$ matrices and let *Q* and *R* be $d\times d$ minor-orthogonal matrices that are independent from *A*, *B* and each other. Then

$$\begin{aligned} {\mathbb {E}}_{Q, R} \left\{ \, \sigma _k((A + Q B R^*)(A + Q B R^*)^*) \,\right\} = \sum _i \frac{\left( {\begin{array}{c}d-i\\ k-i\end{array}}\right) \left( {\begin{array}{c}d-i\\ k-i\end{array}}\right) }{\left( {\begin{array}{c}d\\ k-i\end{array}}\right) \left( {\begin{array}{c}d\\ k-i\end{array}}\right) } \sigma _i(AA^*)\sigma _{k-i}(BB^*). \end{aligned}$$

##### Proof

Let $|U | = k$. Then by Theorem 2.2 we have

$$\begin{aligned}{}[(A + Q B R^*)(A + Q B R^*)^*]_{U, U} = \sum _{|V |=k} [A + Q B R^*]_{U, V} [(A + Q B R^*)^*]_{V, U}. \end{aligned}$$

By Theorem 2.8,

$$\begin{aligned}{}[A + Q B R^*]_{U, V} = \sum _{i=0}^{k} \sum _{|W |=|Z |=i} (-1)^{\Vert W\Vert _1 + \Vert Z\Vert _1} [A]_{W(U), Z(V)} [Q B R^*]_{\overline{W}(U), \overline{Z}(V)} \end{aligned}$$

and

$$\begin{aligned}&[(A + Q B R^*)^*]_{V, U} \\&\quad = \sum _{i=0}^{k} \sum _{|W' |=|Z' |=i} (-1)^{\Vert W'\Vert _1 + \Vert Z'\Vert _1} [A^*]_{W'(V), Z'(U)} [(Q B R^*)^*]_{\overline{W'}(V), \overline{Z'}(U)}. \end{aligned}$$

Applying Lemma 2.13 then gives

$$\begin{aligned}&{\mathbb {E}}_{Q, R} \left\{ \, [Q B R^*]_{\overline{W}(U), \overline{Z}(V)} [(Q B R^*)^*]_{\overline{W'}(V), \overline{Z'}(U)} \,\right\} \\&\quad = \frac{\delta _{ \{ \overline{W'}(V) = \overline{Z}(V) \} } \delta _{ \{ \overline{W}(U) = \overline{Z'}(U) \} } }{\left( {\begin{array}{c}d\\ k-i\end{array}}\right) \left( {\begin{array}{c}d\\ k-i\end{array}}\right) } \sigma _{k-i}(BB^*) \\&\quad = \frac{\delta _{ \{ W' = Z \} } \delta _{ \{ W = Z' \} } }{\left( {\begin{array}{c}d\\ k-i\end{array}}\right) \left( {\begin{array}{c}d\\ k-i\end{array}}\right) } \sigma _{k-i}(BB^*) \end{aligned}$$

where again we use the fact that $A(X) = B(X)$ if and only if $A = B$. Hence

$$\begin{aligned}&{\mathbb {E}}_{Q, R} \left\{ \, [(A + Q B R^*)(A + Q B R^*)^*]_{U, U} \,\right\} \\ {}&= \frac{1}{\left( {\begin{array}{c}d\\ k-i\end{array}}\right) }\frac{1}{\left( {\begin{array}{c}d\\ k-i\end{array}}\right) }\sigma _{k-i}(BB^*) \sum _{|V | = k} \sum _{i=0}^{k} \sum _{|W |=|Z |=i} [A]_{W(U), Z(V)} [A^*]_{Z(V), W(U)}. \end{aligned}$$

Similar to Theorem 2.10, we have

$$\begin{aligned} \sum _{|U |=|V |=k} \sum _{|W |=|Z |=i} [A]_{W(U), Z(V)} [A^*]_{Z(V), W(U)}&= \theta _{i, k} \sum _{|S | = |T | = i} [A]_{S, T} [A^*]_{T, S} \\&= \theta _{i, k} \sum _{|S |= i} [AA^*]_{S, S} \\&= \theta _{i, k} \sigma _i(AA^*), \end{aligned}$$

where $\theta _{i, k}$ is the number of ways to complete *S* to a set of size *k* *and* complete *T* to a set of size *k*. Since $S \subseteq [d]$ and $T \subseteq [d]$, this is precisely $$\left( {\begin{array}{c}d-i\\ k-i\end{array}}\right) \left( {\begin{array}{c}d-i\\ k-i\end{array}}\right) $$, completing the proof. $\square$

## Real rootedness of the asymmetric additive convolution

We will use the theory of stable polynomials to prove Theorem 1.9 (see e.g. [24] for an introduction). Stability is a multivariate generalization of real-rootedness which is preserved under a rich and well-understood class of linear transformations, and our approach is to realize  as a univariate restriction of a multivariate stable polynomial. For this theorem, we will require Hurwitz stable polynomials. We recall that a multivariate polynomial $p (z_{1}, \dots , z_{m}) \in \mathrm{IR}^{}[z_{1}, \dots , z_{m}]$ is *Hurwitz stable* if it is identically zero or if whenever the real part of $z_{i}$ is positive for all *i*, $p (z_{1}, \dots , z_{m}) \not = 0$.

Instead of proving Theorem 1.9 directly, we prove the following theorem from which it follows by substituting $-x$ for *x*. We define ${\mathbb {P}}^{-} (d)$ to be the subset of polynomials in ${\mathbb {P}} (d)$ having only nonpositive roots and ${\mathbb {P}}^{-}$ to be the union over ${\mathbb {P}}^{-} (d)$ for $d \ge 1$.

### Theorem 3.1

Let

$$\begin{aligned} p (x) = \sum _{i=0}^{d} x^{d-i} a_{i} \quad \text {and} \quad q (x) = \sum _{i=0}^{d} x^{d-i} b_{i} \end{aligned}$$

be in ${\mathbb {P}}^{-} (d)$. Then,

$$\begin{aligned} r (x) {\mathop {=}\limits ^{\mathrm {def}}}\sum _{k=0}^{d} x^{d-k} \sum _{i+j = k} \left( \frac{(d-i)! (d-j)!}{d! (d-i-j)!} \right) ^{2} a_{i} b_{j} \end{aligned}$$

is also in ${\mathbb {P}}^{-} (d)$.

We will use the following result to prove that a polynomial is in ${\mathbb {P}}^{-}$.

### Lemma 3.2

Let *r*(*x*) be a polynomial such that $h (x,y) = r (xy)$ is Hurwitz stable. Then, $r \in {\mathbb {P}}^{-}$.

### Proof

Let $\zeta$ be any root of *r*. If $\zeta$ is neither zero or negative, then it has a square root with positive real part. Setting both *x* and *y* to this square root would contradict the Hurwitz stability of *h*. $\square$

We will prove that *r*(*x*) is in ${\mathbb {P}}^{-}$ by constructing a Hurwitz stable polynomial and applying Lemma 3.2. To this end, we need a few tools for constructing Hurwitz stable polynomials.

The first is elementary.

### Claim 3.3

If $p (x) \in {\mathbb {P}}^{-}$, then the polynomial $f (x,y) = p (xy)$ is Hurwitz stable.

### Proof

If both *x* and *y* have positive real part, then *xy* cannot be a nonpositive real, and thus cannot be a root of *p*. $\square$

The second tool is the following result of Borcea and Brändén, which is a consequence of Corollary 5.9 of [25].

### Proposition 3.4

(Polarization) Let

$$\begin{aligned} p (x,y) = \sum _{i=0}^{d}\sum _{j=0}^{d} c_{i,j} x^{i} y^{j} \end{aligned}$$

be a Hurwitz stable polynomial. For each integer *i*, let $\sigma _{i}^{x}$ be the *i*th elementary symmetric polynomial in the variables $x_{1}, \dots , x_{d}$, and let $\sigma _{i}^{y}$ be the corresponding polynomial in $y_{1}, \dots , y_{d}$. Then, the polynomial

$$\begin{aligned} P (x_{1}, \dots , x_{d}, y_{1}, \dots , y_{d}) {\mathop {=}\limits ^{\mathrm {def}}}\sum _{i=0}^{d}\sum _{j=0}^{d} c_{i,j} \frac{\sigma _{i}^{x} \sigma _{j}^{y}}{\left( {\begin{array}{c}d\\ i\end{array}}\right) \left( {\begin{array}{c}d\\ j\end{array}}\right) } \end{aligned}$$

is Hurwitz stable.

The polynomial *P* is called the *polarization* of *p*. We remark that $P (x, \dots ,x, y, \dots , y) = p (x,y)$.

The last result we need is due to Lieb and Sokal [26] (see also [27, Theorem 8.4]).

### Theorem 3.5

Let $P (z_{1}, \dots , z_{d})$ and $Q (z_{1}, \dots , z_{d})$ be Hurwitz stable polynomials. Let $D_{i}^{z}$ denote the derivative with respect to $z_{i}$. Then,

$$\begin{aligned} Q (D_{1}^{z}, \dots , D_{d}^{z}) P (z_{1}, \dots , z_{d}) \end{aligned}$$

is Hurwitz stable.

### Proof of Theorem 3.1

Define $f (x,y) = p (xy)$ and $g (x,y) = (xy)^{d} q (1/xy)$. Let $F (x_{1}, \dotsc , x_{d}, y_{1}, \dotsc , y_{d})$ be the polarization of *f*(*x*, *y*) in the variables $x_{1}, \dots , x_{d}, y_{1}, \dots , y_{d}$. Let $G (x_{1}, \dots , x_{d}, y_{1}, \dots , y_{d})$ be the analogous polarization of *g*(*x*, *y*).

Let $\sigma _{i}^{x}$ be the *i*th elementary symmetric function in $x_{1}, \dotsc , x_{d}$, and let $\delta _{i}^{x}$ be the *i*th elementary symmetric function in $D^{x}_{1}, \dotsc , D^{x}_{d}$. Define $\sigma _{i}^{y}$ and $\delta _{i}^{y}$ analogously.

Then,

$$\begin{aligned} F (x_{1}, \dotsc , x_{d}, y_{1}, \dots , y_{d}) = \sum _{i=0}^{d} a_{i} \frac{\sigma _{d-i}^{x} \sigma _{d-i}^{y} }{\left( {\begin{array}{c}d\\ i\end{array}}\right) ^{2}}, \end{aligned}$$

and

$$\begin{aligned} G (D^{x}_{1}, \dotsc , D^{x}_{d}, D^{y}_{1}, \dotsc , D^{y}_{d}) = \sum _{i=0}^{d} b_{i} \frac{\delta _{i}^{x} \delta _{i}^{y} }{\left( {\begin{array}{c}d\\ i\end{array}}\right) ^{2}}. \end{aligned}$$

Define

$$\begin{aligned}&H (x_{1}, \dotsc , x_{d}, y_{1}, \dots , y_{d}) \\&\quad = G (D^{x}_{1}, \dotsc , D^{x}_{d}, D^{y}_{1}, \dotsc , D^{y}_{d}) F (x_{1}, \dotsc , x_{d}, y_{1}, \dots , y_{d}). \end{aligned}$$

We know from Theorem 3.5 that *H* is Hurwitz stable. Define

$$\begin{aligned} h (x,y) = H (x, \dots , x, y, \dots , y). \end{aligned}$$

It is immediate that *h* is Hurwitz stable too. We will prove that $h (x,y) = r (xy)$, which by Lemma 3.2 implies that *r* is in ${\mathbb {P}}^{-}$.

It will be convenient to know that

$$\begin{aligned} \delta ^{x}_{i} \sigma ^{x}_{j} = {\left\{ \begin{array}{ll} \left( {\begin{array}{c}d+i-j\\ i\end{array}}\right) \sigma ^{x}_{j-i}&{} \hbox { if}\ i \le j \\ 0 &{} \text {otherwise}. \end{array}\right. } \end{aligned}$$

We may now compute

$$\begin{aligned} H (x_{1},\dotsc x_{d}, y_{1}, \dots , y_{d})&= \sum _{i=0}^{d} b_{i} \frac{\delta _{i}^{x} \delta _{i}^{y} }{\left( {\begin{array}{c}d\\ i\end{array}}\right) ^{2}} \sum _{j=0}^{d} a_{j} \frac{\sigma _{d-j}^{x} \sigma _{d-j}^{y} }{\left( {\begin{array}{c}d\\ j\end{array}}\right) ^{2}} \\&= \sum _{i=0}^{d} \sum _{j : i \le d-j} \frac{ b_{i}}{\left( {\begin{array}{c}d\\ i\end{array}}\right) ^{2}} \frac{ a_{j}}{\left( {\begin{array}{c}d\\ j\end{array}}\right) ^{2}} \delta _{i}^{x} \delta _{i}^{y} \sigma _{d-j}^{x} \sigma _{d-j}^{y} \\&= \sum _{i +j \le d} \frac{a_{j} b_{i}}{\left( {\begin{array}{c}d\\ i\end{array}}\right) ^{2} \left( {\begin{array}{c}d\\ j\end{array}}\right) ^{2}} \delta _{i}^{x} \delta _{i}^{y} \sigma _{d-j}^{x} \sigma _{d-j}^{y} \\&= \sum _{i +j \le d} \frac{a_{j} b_{i}}{\left( {\begin{array}{c}d\\ i\end{array}}\right) ^{2} \left( {\begin{array}{c}d\\ j\end{array}}\right) ^{2}} \left( {\begin{array}{c}d+i - (d-j) \\ i\end{array}}\right) ^{2} \sigma _{d-i-j}^{x} \sigma _{d-i-j}^{y} \\&= \sum _{i +j \le d} a_{j} b_{i} \frac{\left( {\begin{array}{c}i+j\\ i\end{array}}\right) ^{2}}{\left( {\begin{array}{c}d\\ i\end{array}}\right) ^{2} \left( {\begin{array}{c}d\\ j\end{array}}\right) ^{2}} \sigma _{d-i-j}^{x} \sigma _{d-i-j}^{y} \\&= \sum _{k = 0}^{d} \sum _{i + j = k} a_{j} b_{i} \frac{\left( {\begin{array}{c}k\\ i\end{array}}\right) ^{2}}{\left( {\begin{array}{c}d\\ i\end{array}}\right) ^{2} \left( {\begin{array}{c}d\\ j\end{array}}\right) ^{2}} \sigma _{d-k}^{x} \sigma _{d-k}^{y}. \end{aligned}$$

So,

$$\begin{aligned} h (x, y)&= \sum _{k = 0}^{d} \sum _{i + j = k} a_{j} b_{i} \left( \frac{\left( {\begin{array}{c}d\\ k\end{array}}\right) \left( {\begin{array}{c}k\\ i\end{array}}\right) }{\left( {\begin{array}{c}d\\ i\end{array}}\right) \left( {\begin{array}{c}d\\ j\end{array}}\right) } \right) ^{2} x^{d-k} y^{d-k}. \\&= \sum _{k = 0}^{d} \sum _{i + j = k} a_{j} b_{i} \left( \frac{ (d-i) ! (d-j) ! }{ d! (d-i-j) ! } \right) ^{2} x^{d-k} y^{d-k}. \end{aligned}$$

So, $r (xy) = h (x,y)$ and therefore must have only nonpositive real roots. $\square$

## Transform bounds

In this section we prove Theorems 1.12, 1.13, and 1.14. All of our transform bounds are proved using the following lemma. It allows us to pinch together two of the roots of a polynomial without changing the value of the Cauchy transform at a particular point. Through judicious use of this lemma, we are able to reduce statements about arbitrary polynomials to statements about polynomials with just one root.

### Lemma 4.1

(Pinching) Let $\alpha > 0$, $d \ge 2$, and let $p (x) \in {\mathbb {P}} (d)$ have at least two distinct roots. Write $p (x) = \prod _{i=1}^{d} (x - \lambda _{i})$ where $\lambda _{1} \ge \lambda _{2} \ge \dots \ge \lambda _{d}$ and $\lambda _{1} > \lambda _{k}$ for some *k*. Then there exist real $\mu$ and $\rho$ so that $p (x) = {\widehat{{p}}}(x) + {\widetilde{{p}}}(x)$, where

$$\begin{aligned}&{\widetilde{{p}}}(x) = (x- \mu )^{2} \prod _{i \not \in \left\{ 1,k \right\} } (x - \lambda _{i}) \in {\mathbb {P}} (d) \quad \text {and} \\&\quad {\widehat{{p}}}(x) = (x- \rho ) \prod _{i \not \in \left\{ 1,k \right\} } (x - \lambda _{i}) \in {\mathbb {P}} (d-1), \end{aligned}$$

and

- $$\mathsf {maxroot} \left( U_{\alpha } {\widetilde{{p}}} \right) = \mathsf {maxroot} \left( U_{\alpha } {\widehat{{p}}} \right) = \mathsf {maxroot} \left( U_{\alpha } p \right) $$,
- $$\lambda _{1}> \mu > \lambda _{k}$$, and
- $\rho > \lambda _{1}$. In particular, if $d \ge 3$ then ${\widehat{{p}}}$ has at least two distinct roots.

### Proof

Let $t = \mathsf {maxroot} \left( U_{\alpha } p \right)$ and set

$$\begin{aligned} \mu = t - \frac{2}{1/(t - \lambda _{1}) + 1/(t - \lambda _{k})}. \end{aligned}$$

We have chosen $\mu$ so that

$$\begin{aligned} \frac{2}{t - \mu } = \frac{1}{t - \lambda _{1}} + \frac{1}{t - \lambda _{k}}, \end{aligned}$$

which implies

$$\begin{aligned} \frac{D{\widetilde{{p}}}(t)}{{\widetilde{{p}}}(t)} = \frac{Dp (t)}{p (t)} = \frac{1}{\alpha }, \end{aligned}$$

and thus $\mathsf {maxroot} \left( U_{\alpha } {\widetilde{{p}}} \right) = t$. Our choice of $\mu$ also guarantees that $t - \mu$ is the harmonic average of $t - \lambda _{1}$ and $t - \lambda _{k}$. Thus, $\mu$ must lie strictly between $\lambda _{1}$ and $\lambda _{k}$, which implies part *b*. As the harmonic mean of distinct numbers is less than their average, $t - \mu < (1/2) (2t - (\lambda _{1} + \lambda _{k}))$, which implies that

15 $$\begin{aligned} \mu > (\lambda _{1} + \lambda _{k})/2. \end{aligned}$$

We have

$$\begin{aligned} {\widehat{{p}}}(x) = p (x) - {\widetilde{{p}}}(x) = \left( (2 \mu - (\lambda _{1} + \lambda _{k} )) x - (\mu ^{2} - \lambda _{1} \lambda _{k}) \right) \prod _{i \not \in \left\{ 1,k \right\} } (x-\lambda _{i}). \end{aligned}$$

This and inequality (15) imply that ${\widehat{{p}}}(x) \in {\mathbb {P}} (d-1)$. As $U_{\alpha }$ is linear, we also have $(U_{\alpha } {\widehat{{p}}}) (t) = 0$. To finish the proof of part *a*, we need to show that the maximum root of $U_{\alpha } {\widehat{{p}}}$ is less than *t*. The one root of ${\widehat{{p}}}$ that is not a root of *p* is

$$\begin{aligned} \rho {\mathop {=}\limits ^{\mathrm {def}}}\frac{\mu ^{2} - \lambda _{1} \lambda _{k}}{2 \mu - (\lambda _{1} + \lambda _{k} )}. \end{aligned}$$

To see that $t > \rho$, compute

$$\begin{aligned} t- \rho = \frac{(\lambda _{1} - \mu ) (\mu -\lambda _{k})}{2 \mu - \lambda _{1} - \lambda _{k}}, \end{aligned}$$

which we know is greater than 0 because of (15) and the fact that $\mu$ is between $\lambda _{1}$ and $\lambda _{k}$. This completes the proof of part *a*.

To prove part *c*, note that $2 \mu - (\lambda _{1} + \lambda _{k}) > 0$, and

$$\begin{aligned} (2 \mu - (\lambda _{1} + \lambda _{k})) (\rho - \lambda _{1}) = \mu ^{2} - 2 \lambda _{1} \mu + \lambda _{1}^{2} = (\mu -\lambda _{1})^{2} > 0. \end{aligned}$$

$$\square $$

The following lemma provides one of the facts we exploit about the decomposition $p(x) = {\widetilde{{p}}}(x) + {\widehat{{p}}}(x)$

### Lemma 4.2

Let *f*, *g*, *h* be real rooted polynomials with positive leading coefficients such that $f = g + h$. Then

16 $$\begin{aligned} \mathsf {maxroot} \left( f \right) \le \max \left\{ \mathsf {maxroot} \left( g \right) , \mathsf {maxroot} \left( h \right) \right\} \end{aligned}$$

with equality if and only if

17 $$\begin{aligned} \mathsf {maxroot} \left( f \right) = \mathsf {maxroot} \left( g \right) = \mathsf {maxroot} \left( h \right) . \end{aligned}$$

### Proof

Note that equality in (17) clearly implies equality in (16). Now, assume by way of contradiction that (16) is false, and let $x =\mathsf {maxroot} \left( f \right)$. Since *g* and *h* have positive leading terms, $g(x), h(x) > 0$. Thus, $f(x) = g(x) + h(x) > 0$, a contradiction . $\square$

### Symmetric additive convolution

We now prove the upper bound on the *R*-transform of .

#### Theorem 4.3

(Restatement of Theorem 1.12) For $p,q \in {\mathbb {P}} (d)$ and $\alpha >0$,with equality only if *p*(*x*) or *q*(*x*) has the form $(x-\lambda )^{d}$.

Theorem 1.2 and Lemma 1.16 tell us that if $q (x) \in {\mathbb {P}} (d)$ and $p (x) = x^{d-1}$, then . As $\mathsf {maxroot} \left( U_{\alpha } x^{d-1} \right) = (d-1) \alpha$, the following lemma may be viewed as a restriction of Theorem 4.3 to the case that $q (x) = x^{d-1}$.

#### Lemma 4.4

For $$\alpha \ge 0$$, $$d \ge 2$$, and $$p \in {\mathbb {P}} (d)$$,

18 $$\begin{aligned} \mathsf {maxroot} \left( U_{\alpha } D p \right) \le \mathsf {maxroot} \left( U_{\alpha } p \right) - \alpha . \end{aligned}$$

with equality if and only if $p = (x - \lambda )^d$ for some real number $\lambda$.

#### Proof

If $p = (x-\lambda )^{d}$, then $\mathsf {maxroot} \left( U_{\alpha } p \right) = \lambda + d\alpha$ and $\mathsf {maxroot} \left( U_{\alpha } Dp \right) = \lambda + (d-1)\alpha$, giving equality in (18).

We now prove the rest of the lemma by induction on *d*, with $d = 2$ being the base case. To establish the lemma in the case that $d = 2$ and *p* has roots $\lambda _{1} > \lambda _{2}$, note that

$$\begin{aligned} r {\mathop {=}\limits ^{\mathrm {def}}}\mathsf {maxroot} \left( U_{\alpha } Dp \right) + \alpha = 2 \alpha + (\lambda _{1} + \lambda _{2})/2 \end{aligned}$$

and

$$\begin{aligned} U_{\alpha } p(x) = x^2 - (\lambda _{1} + \lambda _{2} + 2 \alpha ) x + \alpha (\lambda _{1} + \lambda _{2}) + \lambda _{1} \lambda _{2}. \end{aligned}$$

As this polynomial has a positive leading term, the fact that $\mathsf {maxroot} \left( U_{\alpha } p \right) > r$ follows from

$$\begin{aligned} U_{\alpha } p(r) = - (\lambda _{1} - \lambda _{2})^2 / 4 < 0. \end{aligned}$$

For a real rooted polynomial *p*, define

$$\begin{aligned} \phi (p) = \mathsf {maxroot} \left( U_{\alpha } p \right) - \alpha - \mathsf {maxroot} \left( U_{\alpha } Dp \right) . \end{aligned}$$

We will prove by induction on *d* that $\phi (p) > 0$ for all polynomials $p \in {\mathbb {P}} (d)$ that have more than one root.

Assume by way of contradiction that there exists a monic (without loss of generality, since $\phi$ is independent of scaling) polynomial $p \in {\mathbb {P}} (d)$ with at least two distinct roots for which $\phi (p) \le 0$. Let $[-R, R]$ be an interval containing all of the roots of *p*, and define ${\mathbb {P}} (d)[-R, R]$ to be all monic polynomials in ${\mathbb {P}} (d)$ with all roots in this interval. Since $[-R,R]^{d}$ is a compact set and $\phi$ is a continuous function of the roots of *p*, there is a monic polynomial $p_0 \in {\mathbb {P}} (d)[-R, R]$ at which $\phi$ obtains its minimum. Let $p_0$ be such a polynomial, so $\phi (p_0) \le \phi (p) \le 0$. We may assume that $p_0$ has at least two distinct roots, because it is true if $\phi (p_0) < 0$ whereas if $\phi (p_0) = 0$ we may assume $p_0 = p$.

Lemma 4.1 implies that there exist polynomials ${\widehat{{p}}}$ and ${\widetilde{{p}}}$ with $p_0 = {\widehat{{p}}}+ {\widetilde{{p}}}$ such that

1. ${\widehat{{p}}}$ and ${\widetilde{{p}}}$ have positive first coefficients
2. ${\widehat{{p}}}$ has degree $d-1$, and if $d \ge 3$ it has at least two distinct roots.
3. $${\widetilde{{p}}}\in {\mathbb {P}} (d)[-R, R]$$.
4. $$\mathsf {maxroot} \left( U_{\alpha } {\widetilde{{p}}} \right) = \mathsf {maxroot} \left( U_{\alpha } {\widehat{{p}}} \right) = \mathsf {maxroot} \left( U_{\alpha } p_0 \right) $$

By linearity we have

$$\begin{aligned} {U_{\alpha } D{\widetilde{{p}}}} + {U_{\alpha } D{\widehat{{p}}}} = {U_{\alpha } Dp_0}. \end{aligned}$$

If $$\mathsf {maxroot} \left( U_{\alpha } Dp_0 \right) \le \mathsf {maxroot} \left( U_{\alpha } D{\widehat{{p}}} \right) $$, then

$$\begin{aligned} \phi (p_0)&= \mathsf {maxroot} \left( U_{\alpha } p_0 \right) - \alpha - \mathsf {maxroot} \left( U_{\alpha } Dp_0 \right) \\&\ge \mathsf {maxroot} \left( U_{\alpha } {\widehat{{p}}} \right) - \alpha - \mathsf {maxroot} \left( U_{\alpha } D{\widehat{{p}}} \right) \\&= \phi ({\widehat{{p}}}) \\&> 0, \end{aligned}$$

by the inductive hypothesis, as ${\widehat{{p}}}$ has degree $d-1$. As this would contradict our assumption that $\phi (p) \le 0$, we may assume $\mathsf {maxroot} \left( U_{\alpha } Dp_0 \right) > \mathsf {maxroot} \left( U_{\alpha } D{\widehat{{p}}} \right)$ and apply Lemma 4.2 to conclude $\mathsf {maxroot} \left( U_{\alpha } Dp_0 \right) < \mathsf {maxroot} \left( U_{\alpha } D{\widetilde{{p}}} \right)$. This implies

$$\begin{aligned} \phi (p_0)&= \mathsf {maxroot} \left( U_{\alpha } p_0 \right) - \alpha - \mathsf {maxroot} \left( U_{\alpha } Dp_0 \right) \\&> \mathsf {maxroot} \left( U_{\alpha } {\widehat{{p}}} \right) - \alpha - \mathsf {maxroot} \left( U_{\alpha } D{\widetilde{{p}}} \right) \\&= \phi ({\widetilde{{p}}}) , \end{aligned}$$

contradicting the minimality of $p_0$. Thus, we may conclude that $\phi (p) > 0$ for all $p \in {\mathbb {P}} (d)$ with at least two roots.

$$\square $$

#### Lemma 4.5

For $\alpha \ge 0$, $q = (x- \lambda )^{d}$ for some real $\lambda$ and $p \in {\mathbb {P}} (d)$,

#### Proof

We can prove this either by manipulating the identity in (1) and those following it, or by going through the defintion (1.1). To pursue the latter route, let *A* be a Hermitian matrix whose characteristic polynomials is *p* and let $B = \lambda I$. We then haveThus,On the other hand, $\mathsf {maxroot} \left( U_{\alpha } q \right) = \lambda + \alpha d$. $\square$

#### Lemma 4.6

If $p \in {\mathbb {P}} (d)$ for $d \ge 3$ and *Dp* has just one root, then *p* has just one root.

#### Proof

If $D p = (x - \lambda )^{d-1}$, then *p* can be written in the form $(x - \lambda )^{d} + c$ for some constant *c*. If $d \ge 3$ and *c* were a constant other than zero, then this polynomial would have at least two complex roots. $\square$

Our proof of Theorem 4.3 will be very similar to our proof of Lemma 4.4.

#### Proof of Theorem 4.3

Lemma 4.5 proves the theorem in the case that either *p* or *q* can be written in the form $(x - \lambda )^d$. So, we will prove that if neither *p* nor *q* is of the form $(x - \lambda )^d$ then19We will prove this by induction on *d*, the maximum degree of *p* and *q*. The base case $d=1$ is handled by Lemma 4.5. Assume $d\ge 2$ and fix a polynomial $q\in {\mathbb {P}} (d)$ with at least two roots. For any polynomial *p* in ${\mathbb {P}} (d)$, defineAs before, assume for contradiction that there exists a monic polynomial *p* with at least two roots for which $\phi (p) \le 0$. Let $[-R, R]$ be an interval containing the roots of *p* and let $p_0$ minimize $\phi$ over all monic polynomials whose roots are contained in this interval. We may assume that $p_0$ has at least two roots because Lemma 4.5 says it must if $\phi (p_0) < 0$, and otherwise we may take $p_0 = p$.

Thus, we can apply Lemma 4.1 to $p_0$ to obtain polynomials ${\widehat{{p}}}\in {\mathbb {P}} (d-1)$ and ${\widetilde{{p}}}\in {\mathbb {P}} (d)$ such that

- $p_0 = {\widehat{{p}}}+ {\widetilde{{p}}}$, which by the linearity of  and $U_{\alpha }$ implies
- the roots of ${\widetilde{{p}}}$ lie inside $[-R, R]$, and so $\phi ({\widetilde{{p}}}) \ge \phi (p_0)$; and
- $$\mathsf {maxroot} \left( U_{\alpha } {\widetilde{{p}}} \right) = \mathsf {maxroot} \left( U_{\alpha } {\widehat{{p}}} \right) = \mathsf {maxroot} \left( U_{\alpha } p_0 \right) $$.

By Lemma 1.16As the degree of *Dq* is less than *d*, and *Dq* has at least two distinct roots by Lemma 4.6 we may apply our inductive hypothesis to conclude thatas $\phi (p_0) \le 0$. Thus, property (*a*) above and Lemma 4.2 imply thatAs $\mathsf {maxroot} \left( U_{\alpha } {\widetilde{{p}}} \right) = \mathsf {maxroot} \left( U_{\alpha } p_0 \right)$, this implies $\phi ({\widetilde{{p}}}) < \phi (p_0)$, a contradiction. Thus, (19) holds when both polynomials have at least two roots.

$$\square $$

### Symmetric multiplicative convolution

In this section we prove the following upperbound on the variant of the $S-$transform of , defined in Sect. 1.3.

#### Theorem 4.7

(Restatement of Theorem 1.13) For $p, q\in {\mathbb {P}}^{+} (d)$ having only nonnegative real roots and $w > 0$,with equality only when *p* or *q* has only one distinct root.

We begin by considering the case in which $p = (x-\lambda )^{d}$. We then have that

$$\begin{aligned} \widetilde{{\mathcal {M}}}_{p} \left( z \right) = \sum _{j \ge 1} (\lambda /z)^{j} = \frac{\lambda }{z - \lambda }. \end{aligned}$$

Thus,

$$\begin{aligned} \widetilde{{\mathcal {M}}}^{(-1)}_{p} \left( w \right) = \frac{1+w}{w} \lambda , \end{aligned}$$

and

$$\begin{aligned} \widetilde{{\mathcal {S}}}_{p} \left( w \right) = \lambda . \end{aligned}$$

#### Lemma 4.8

If $\lambda > 0$, $p (x) = (x-\lambda )^{d}$ and $q (x) \in {\mathbb {P}}^{+} (d)$, then for all $w \ge 0$

#### Proof

For $p (x) = (x-\lambda )^{d}$, one may use either the definition (1.4) or Theorem 1.5 to computeAs, $\widetilde{{\mathcal {M}}}_{q (x/\lambda )} \left( \lambda z \right) = \widetilde{{\mathcal {M}}}_{q (x)} \left( z \right)$,$\square$

The finite multiplicative convolution of polynomials of different degrees may be computed by taking the *polar derivative with respect to 0* of the polynomial of higher degree. We recall that the polar derivative at 0 of a polynomial *p* of degree *d* is given by $d p - x Dp$ (see [2, p. 44]).

#### Lemma 4.9

(Degree Reduction for  ) For $p (x) \in {\mathbb {P}} (d)$ and $q (x) \in {\mathbb {P}} (k)$, for $k < d$,

#### Proof

Follows from equation (5) by an elementary computation. $\square$

#### Remark 4.10

If we let *R* be the operation on polynomials in ${\mathbb {P}}^{+} (d)$ that maps *p*(*x*) to $x^{d} p (1/x)$. The polar derivative of a degree *d* polynomial may be expressed in terms of *R* by

$$\begin{aligned} d p - x Dp = R DR p. \end{aligned}$$

In particular, the polar derivative has a discontinuity at $\infty$ that occurs as a root of *DRp* passes 0, causing technical issues when considering polynomials *p*(*x*) with both positive and negative roots (especially given that the root we are concerned in is the largest one). This prevents our proof method (which we chose to highlight the parallel between the additive and multiplicative case) from generalizing to a larger collection of polynomials. We therefore leave the possibility of a generalization as an open problem.

#### Claim 4.11

For $$p = (x-\lambda )^{d}$$,

$$\begin{aligned} x Dp - d p = \lambda d (x- \lambda )^{d-1}. \end{aligned}$$

For $$p \in {\mathbb {P}}^{+} (d)$$, $$(x D - d)p \in {\mathbb {P}}^{+} (d-1)$$ and

20 $$\begin{aligned} \mathsf {maxroot} \left( p \right) \ge \mathsf {maxroot} \left( x Dp - d p \right) . \end{aligned}$$

In the special case of $p\in {\mathbb {P}}^{+} (2)$ with distinct roots, strict inequality holds:

21 $$\begin{aligned} \mathsf {maxroot} \left( p \right) > \mathsf {maxroot} \left( xDp - 2p \right) . \end{aligned}$$

#### Proof

The first part is a simple calculation. Inequality (20) follows from the fact that $p \in {\mathbb {P}}^{+} (d)$ implies that $R p \in {\mathbb {P}}^{+} (d)$ and the fact that the roots of $DR p$ interlace those of *Rp*. To see that $(x D - d)p \in {\mathbb {P}}^{+} (d-1)$, observe that its lead coefficient is positive.

The last claim follows by noting that *Rp* is quadratic polynomial with distinct roots and so *DRp* strictly interlaces *Rp*. $\square$

As we did with the symmetric additive convolution, we relate the $\widetilde{{\mathcal {M}}}$-transform to the maximum root of a polynomial. We have

$$\begin{aligned} \widetilde{{\mathcal {M}}}_{p} \left( z \right) = w \quad \iff \quad \mathsf {maxroot} \left( \left( 1 - \frac{x D}{d (w+1)} \right) p (x) \right) = 0. \end{aligned}$$

We therefore define the operator $V_{w}$ by

$$\begin{aligned} V_{w} p (x)= \left( 1 - \frac{x D}{d (w+1)} \right) p (x), \end{aligned}$$

which gives

$$\begin{aligned} \widetilde{{\mathcal {M}}}_{p} \left( z \right) = w \quad \iff \quad \mathsf {maxroot} \left( V_{w} p \right) = z. \end{aligned}$$

Note that the polar derivative is $d V_{0}$.

Our proof of Theorem 4.7 will also employ the following consequence of Lemma 4.1.

#### Corollary 4.12

Let $w > 0$, $d \ge 2$, and let $p (x) \in {\mathbb {P}}^{+} (d)$ have at least two distinct roots. Then there exist ${\widetilde{{p}}}\in {\mathbb {P}}^{+} (d)$ and ${\widehat{{p}}}\in {\mathbb {P}}^{+} (d-1)$ so that $p (x) = {\widehat{{p}}}(x) + {\widetilde{{p}}}(x)$, the largest root of ${\widetilde{{p}}}$ is at most the largest root of *p*,

$$\begin{aligned} \mathsf {maxroot} \left( V_{w} p \right) = \mathsf {maxroot} \left( V_{w} {\widetilde{{p}}} \right) = \mathsf {maxroot} \left( V_{w} {\widehat{{p}}} \right) , \end{aligned}$$

and if $d \ge 3$ then ${\widehat{{p}}}$ has two distinct roots.

#### Proof

To derive this from Lemma 4.1, let $t = \mathsf {maxroot} \left( V_{w} p \right)$ and set

$$\begin{aligned} \alpha = \frac{t}{d (w+1)}. \end{aligned}$$

The polynomials ${\widetilde{{p}}}$ and ${\widehat{{p}}}$ constructed in Lemma 4.1 now satisfy

$$\begin{aligned} \mathsf {maxroot} \left( U_{\alpha } {\widehat{{p}}} \right) = \mathsf {maxroot} \left( U_{\alpha } {\widetilde{{p}}} \right) = t = \mathsf {maxroot} \left( V_{w} {\widetilde{{p}}} \right) = \mathsf {maxroot} \left( V_{w} {\widehat{{p}}} \right) , \end{aligned}$$

as desired. $$\square $$

#### Proof of Theorem 4.7

We proceed by induction on *d*, the maximum degree of *p* and *q*. The theorem is true for $d=1$ by Lemma 4.8. As we have already shown that equality holds when one of *p* or *q* has just one root, we need to show that when both *p* and *q* have at least two distinct roots:Fix $q\in {\mathbb {P}}^{+} (d)$ with at least two distinct roots, and for $p \in {\mathbb {P}}^{+}$ define:As before, we assume (for contradiction) that there exists a monic *p* with two distinct roots and $\phi (p) \le 0$. Choose an interval [0, *R*] containing the roots of *p*, and let $p_0$ minimize $\phi$ over all degree *d* monic polynomials with roots in this interval. Observe that we can choose $p_0$ having two distinct roots: if $\phi (p_0)<0$ this is implied by Lemma 4.8 and if $\phi (p_0)=0$ we can take $p=p_0$. Thus we may apply Corollary 4.12 to obtain polynomials ${\widetilde{{p}}}\in {\mathbb {P}}^{+} (d)$ and ${\widehat{{p}}}\in {\mathbb {P}}^{+} (d-1)$ with $p_0 = {\widetilde{{p}}}+ {\widehat{{p}}}$,

22 $$\begin{aligned} \mathsf {maxroot} \left( V_{w} p_0 \right) = \mathsf {maxroot} \left( V_{w} {\widetilde{{p}}} \right) = \mathsf {maxroot} \left( V_{w} {\widehat{{p}}} \right) , \end{aligned}$$

and $\mathsf {maxroot} \left( {\widetilde{{p}}} \right) \le \mathsf {maxroot} \left( p_0 \right)$. By Lemma 4.2, we havewith equality only if all three are equal. However, noting that ${\widehat{{p}}}$ and $(xD-d)q= -RDRq$ have two distinct roots whenever $d\ge 3$:since $\phi (p_0)\le 0$. Since at least one of the inequalities above is strict for all $d\ge 2$, we must have , which implies $\phi ({\widetilde{{p}}})<\phi (p_0)$, contradicting the minimality of $p_0$. $\square$

### Asymmetric additive convolution

In this section we prove the rectangular analogue of Theorem 1.12.

#### Theorem 4.13

(Restatement of Theorem1.14) Let *p*(*x*) and *q*(*x*) be in ${\mathbb {P}}^{+} (d)$ for $d \ge 1$. Then for all $\alpha \ge 0$,with equality if and only if *p* or *q* equals $x^{d}$.

We remark that if $q (x) = x^{d}$, then , and

$$\begin{aligned} U_{\alpha } {\mathbb {S}}q = U_{\alpha } x^{2d} = x^{2d-1} (x - 2 d \alpha ), \end{aligned}$$

so

$$\begin{aligned} \mathsf {maxroot} \left( U_{\alpha } {\mathbb {S}}q \right) = 2 \alpha d. \end{aligned}$$

This is why Theorem 4.13 holds with equality when $q (x) = x^{d}$.

The following lemma tells us that it suffices to prove Theorem 4.13 in the case that $\alpha = 1$.

#### Lemma 4.14

For a real-rooted polynomial *p*(*x*),

$$\begin{aligned} \mathsf {maxroot} \left( U_{\alpha } p (x) \right) = \frac{1}{\alpha } \mathsf {maxroot} \left( U_{1} p (\alpha x) \right) . \end{aligned}$$

#### Proof

Let $$q (x) = p (\alpha x)$$, so

$$\begin{aligned} U_{1} q (x) = p (\alpha x) - \alpha p' (\alpha x). \end{aligned}$$

Let

$$\begin{aligned} w = \alpha \text {--}\mathsf {max}\left( p (x) \right) = \mathsf {maxroot} \left( U_{\alpha } p \right) \quad \iff \quad p (w) - \alpha p' (w) = 0. \end{aligned}$$

Then,

$$\begin{aligned} (U_{1} q) (w/\alpha ) = p (w) - \alpha p' (w) = 0. \end{aligned}$$

$$\square $$

Our proof of Theorem 4.13 will use the following lemma to pinch together roots of *p* to reduce the analysis to a few special cases.

#### Corollary 4.15

Let $\alpha > 0$, $d \ge 2$, and let $p (x) \in {\mathbb {P}}^{+} (d)$ have at least two distinct roots. Then there exist ${\widetilde{{p}}}\in {\mathbb {P}}^{+} (d)$ and ${\widehat{{p}}}\in {\mathbb {P}}^{+} (d-1)$ so that $p (x) = {\widehat{{p}}}(x) + {\widetilde{{p}}}(x)$, the largest root of ${\widetilde{{p}}}$ is at most the largest root of *p*, ${\widehat{{p}}}$ has a root larger than 0, and

23 $$\begin{aligned} \mathsf {maxroot} \left( U_{\alpha } {\mathbb {S}}{\widetilde{{p}}} \right) = \mathsf {maxroot} \left( U_{\alpha } {\mathbb {S}}{\widehat{{p}}} \right) = \mathsf {maxroot} \left( U_{\alpha } {\mathbb {S}}p \right) \end{aligned}$$

#### Proof

Let $$t = \mathsf {maxroot} \left( U_{\alpha } {\mathbb {S}}p \right) $$, so

$$\begin{aligned} \mathsf {maxroot} \left( (1 - 2 \alpha t D)p \right) = \sqrt{t}. \end{aligned}$$

Apply Lemma 4.1 with $2 \alpha t$ in the place of $\alpha$ to construct the polynomials ${\widetilde{{p}}}$ and ${\widehat{{p}}}$. They satisfy

$$\begin{aligned} \mathsf {maxroot} \left( U_{\alpha } {\widehat{{p}}} \right) = \mathsf {maxroot} \left( U_{\alpha } {\widetilde{{p}}} \right) = \sqrt{t}, \end{aligned}$$

which implies (23). $$\square $$

We will build up to the proof of Theorem 4.13 by first handling three special cases:

- When $p (x) = (x-\lambda )^{d}$ and $q (x) = x^{d-1}$. That is, we consider $Dx D(x-\lambda )^{d}$ (Lemma 4.18).
- When $p (x) \in {\mathbb {P}}^{+} (d)$ and $q (x) = x^{d-1}$. That is, we consider $Dx Dp (x)$ (Lemma 4.19).
- When $p (x) = (x-\lambda )^{d}$ and $q (x) = (x-\mu )^{d}$ (Lemma 4.20).

As with the other convolutions, we may compute the asymetric additive convolution of two polynomials by first applying an operation to the polynomial of higher degree. In this case it is $Dx D$, also known as the “Laguerre Derivitive”.

#### Lemma 4.16

(Degree Reduction for ) Let $p \in {\mathbb {P}}^{+} (d)$ and let $q \in {\mathbb {P}}^{+} (k)$ for $k < d$. Then,

#### Proof

Follows from Theorem 1.8. $\square$

The following characterization of the Laguerre derivitive also follows from Theorem 1.8.

#### Claim 4.17

If $$q (x) = x^{d-1}$$, then

#### Lemma 4.18

For $$\alpha \ge 0$$, $$\lambda \ge 0$$, $$d \ge 2$$ and $$p (x) = (x-\lambda )^{d}$$,

$$\begin{aligned} \mathsf {maxroot} \left( U_{\alpha } {\mathbb {S}}Dx Dp \right) \le \mathsf {maxroot} \left( U_{\alpha } {\mathbb {S}}p \right) - 2 \alpha , \end{aligned}$$

with equality only if $\lambda = 0$.

#### Proof

By Lemma 4.14, it suffices to consider the case of $\alpha = 1$. As ${\mathbb {S}}p (x) = (x^{2} - \lambda )^{d}$,

$$\begin{aligned} U_{1 } {\mathbb {S}}p (x) = (x^{2} - 2 \lambda d - \lambda ) (x^{2} - \lambda )^{d-1}. \end{aligned}$$

So, the largest root of this polynomial is the largest root of

$$\begin{aligned} r_{\lambda } (x) {\mathop {=}\limits ^{\mathrm {def}}}(x^{2} - 2 \lambda d - \lambda ). \end{aligned}$$

We may also compute

$$\begin{aligned} U_{1 } {\mathbb {S}}Dx D(x^{2}- \lambda )^{d} = q_{\lambda } (x) (x^{2} - \lambda )^{d-2}, \end{aligned}$$

where

$$\begin{aligned} q_{\lambda } (x) {\mathop {=}\limits ^{\mathrm {def}}}d x^{4} - 2 d (d-1) x^{3} - (d+1) \lambda x^{2} + 4 (d-1) \lambda x + \lambda ^{2}. \end{aligned}$$

We now prove that

$$\begin{aligned} \mathsf {maxroot} \left( q_{\lambda } \right) \le \mathsf {maxroot} \left( r_{\lambda } \right) - 2, \end{aligned}$$

with equality only if $\lambda = 0$.

We first argue that $q_{\lambda } (x)$ is real rooted. This follows from that fact that it is a factor of $U_{1} {\mathbb {S}}Dx D(x-\lambda )^{d}$. For $\lambda \ge 0$ all of the roots of $Dx D(x-\lambda )^{d}$ are nonnegative, and so applying ${\mathbb {S}}$ to it yields a polynomial with all real roots.

We now compute

$$\begin{aligned} \mathsf {maxroot} \left( r_{\lambda } \right) = d + \sqrt{d^{2} + \lambda }. \end{aligned}$$

Define

$$\begin{aligned} \mu _{\lambda } = d + \sqrt{d^{2} + \lambda } - 2. \end{aligned}$$

Elementary algebra gives

$$\begin{aligned} q_{\lambda } (\mu _{\lambda }) = 4 \lambda - 8 d \sqrt{d^{2} + \lambda } + 8 d^{2} = (2 d - 2 \sqrt{d^{2} + \lambda })^{2}. \end{aligned}$$

So, $q_{\lambda } (\mu _{\lambda }) \ge 0$, with equality only when $\lambda = 0$. With just a little more work, we will show that $\mu _{\lambda }$ is an upper bound on the roots of $q_{\lambda }$ for all $\lambda$.

For $q_{\lambda }$ to have a root larger than $\mu _{\lambda }$, it would have to have two roots larger than $\mu _{\lambda }$. When $\lambda = 0$, the polynomial $q_{\lambda }$ has one root at $\mu _{0}$ and a root at 0 with multiplicity 3. As $q_{\lambda }$ is real rooted for all $\lambda \ge 0$ and the roots of $q_{\lambda }$ are continuous functions of its coefficients, and thus of $\lambda$, we can conclude that for small $\lambda$ all but one of the roots of $q_{\lambda }$ must be near 0. Thus, for sufficiently small $\lambda$, $q_{\lambda }$ can have at most one root greater than $\mu _{\lambda }$, and so it must have none. As the largest root of $q_{\lambda }$ and $\mu _{\lambda }$ are continuous function of $\lambda$, $\mathsf {maxroot} \left( q_{\lambda } \right) > \mu _{\lambda }$ for all sufficiently small $\lambda$. As $q_{\lambda } (\mu _{\lambda }) > 0$ for all $\lambda \ge 0$, we can conclude that $\mathsf {maxroot} \left( q_{\lambda } \right) > \mu _{\lambda }$ for all $\lambda \ge 0$. $\square$

To see that Lemma 4.18 is equivalent Theorem 4.13 in the case of $q = x^{d-1}$, note that for $q (x) = x^{d-1}$,

$$\begin{aligned} U_{\alpha } {\mathbb {S}}q (x) = U_{\alpha } q (x^{2}) = x^{2 (d-1)} - \alpha D x^{2 (d-1)} = x^{2d-3} (x - 2 (d-1) \alpha ). \end{aligned}$$

The equivalence now follows from Claim 4.17 and the fact that the the largest root of this polynomial is $2 (d-1) \alpha$.

#### Lemma 4.19

For $$\alpha \ge 0$$, $$d \ge 2$$ and $$p \in {\mathbb {P}}^{+} (d)$$,

$$\begin{aligned} \mathsf {maxroot} \left( U_{\alpha } {\mathbb {S}}Dx Dp \right) \le \mathsf {maxroot} \left( U_{\alpha } {\mathbb {S}}p \right) - 2 \alpha , \end{aligned}$$

with equality only if $p (x) = x^{d}$.

#### Proof

For every $p \in {\mathbb {P}}^{+}$, define

$$\begin{aligned} \phi (p) = \mathsf {maxroot} \left( U_{\alpha } {\mathbb {S}}p \right) - \mathsf {maxroot} \left( U_{\alpha } {\mathbb {S}}Dx Dp \right) - 2 \alpha . \end{aligned}$$

We will show that $\phi (p) \ge 0$ for every polynomial $p \in {\mathbb {P}}^{+}$ of degree at least 2, with equality only when $p = x^{d}$.

Our proof will be by induction on the degree of *p*. Assume that there exists a polynomial $p \in {\mathbb {P}}^{+} (d)$ with $\phi (p) < 0$, let [0, *R*] be an interval containing the roots of *p*, and let $p_0$ minimize $\phi$ over polynomials with roots in that interval. By Lemma 4.18, $p_0$ must have at least 2 distinct roots, and so we can apply Corollary 4.15 to obtain polynomials ${\widehat{{p}}}$ and ${\widetilde{{p}}}$.

Let $x = \mathsf {maxroot} \left( U_{\alpha } {\mathbb {S}}Dx Dp \right)$. If $d=2$, then ${\widehat{{p}}}$ has degree 1 and so $U_{\alpha } {\mathbb {S}}Dx D{\widehat{{p}}}$ equals the lead coefficient of ${\widehat{{p}}}$, which implies

24 $$\begin{aligned} U_{\alpha } {\mathbb {S}}Dx D{\widehat{{p}}}(x) > 0. \end{aligned}$$

For $d \ge 3$, we can then assume by induction that $\phi ({\widehat{{p}}}) > 0$, which then implies (24) as well. Combining this with Lemma 4.2, we get that $\phi (p_0) > \phi ({\widetilde{{p}}})$ which contradicts the minimality of $p_0$.

$$\square $$

In Sect. 4.4, we establish the following special case of Theorem 4.13.

#### Lemma 4.20

For $\lambda , \mu > 0$, and $d \ge 1$, let $p (x) = (x - \lambda )^{d}$ and $q (x) = (x-\mu )^{d}$. Then for all $\alpha \ge 0$,

We now use Lemma 4.20 to prove Theorem 4.13 through a variation of the pinching argument employed in the proof of Lemma 4.18.

#### Proof of Theorem 4.13

We will prove this by induction on the maximum degree of *p* and *q*, which we call *d*. Our base case of $d=1$ is handled by Lemma 4.20.

Assume, without loss of generality, that the degree of *p* is at least the degree of *q*. If the degree of *p* is larger than the degree of *q*, then we may prove the hypothesis byLemma 4.18 also tells us that equality is only achieved when $p = x^{d}$.

We now consider the case in which both *p* and *q* have degree *d*. For polynomials *p* and *q* in , defineWe will prove that $\phi (p,q) \ge 0$ for all such polynomials.

Assume (for contradiction) that there exist polynomials *p*, *q* with $\phi (p, q) < 0$ and let [0, *R*] be an interval containing the roots of *p* and *q*. Again, $\phi$ is a continuous function (this time on the compact set $[0, R]^{2d}$) so let $p_0, q_0$ be a minimizer. If *both* $p_0$ and $q_0$ have at most 1 distinct root, then Lemma 4.20 implies $\phi (p_0,q_0) \ge 0$, with equality only if one of them equals $x^{d}$ (a contradiction). Hence we can assume without loss of generality that $p_0$ has at least 2 distinct roots and so Corollary 4.12 provides polynomials ${\widehat{{p}}}$ and ${\widetilde{{p}}}$ with

$$\begin{aligned} \mathsf {maxroot} \left( U_{\alpha }{\mathbb {S}}p_0 \right) = \mathsf {maxroot} \left( U_{\alpha } {\mathbb {S}}{\widetilde{{p}}} \right) = \mathsf {maxroot} \left( U_{\alpha } {\mathbb {S}}{\widehat{{p}}} \right) \end{aligned}$$

and by Lemma 4.2which in turn implies $\phi (p_0, q_0) \ge \min { \phi ({\widehat{{p}}}, q_0), \phi ({\widetilde{{p}}}, q_0) }$ with equality if and only if all are equal. Again equality cannot occur, since $\phi (p_0, q_0) < 0$ by assumption and $\phi ({\widehat{{p}}}, q_0) \ge 0$ by the inductive hypothesis and $\phi (p_0, q_0) > \phi ({\widehat{{p}}}, q_0)$ cannot occur for the same reason. But this implies $\phi (p_0, q_0) > \phi ({\widetilde{{p}}}, q_0)$, which contradicts the minimality of the pair $(p_0, q_0)$.

$$\square $$

### Ultraspherical polynomials

This section is devoted to the proof of Lemma 4.20. It is a consequence of the following lemma.

#### Lemma 4.21

For $d \ge 0$ and positive $\lambda$ and $\mu$,

Lemma 4.20 then follows from Theorem 4.3. We will prove Lemma 4.21 by showing that the polynomial on the left is a scaled Chebyshev polynomial of the second kind, and that the polynomial on the right is a Legendre polynomial with the same scaling. We then appeal to known relations between the roots of these polynomials.

The Cheybshev and Legendre polynomials are both Ultraspherical (also called Gegenbauer) polynomials. These are special cases of Jacobi polynomials. It is known that their roots all lie between $-1$ and 1 and that they are symmetric about zero (see Theorem 3.3.1 of [28]).

The Ultraspherical polynomials with parameter $\alpha$ are defined by the generating function

25 $$\begin{aligned} \sum _n C^{(\alpha )}_n(x) t^n = \frac{1}{(1 - 2 x t + t^2)^{\alpha }}. \end{aligned}$$

Two special instances of these polynomials are

1. the Legendre polynomials: $P_d (x) = C^{(1/2)}_d(x)$, and
2. the Chebyshev polynomials of the second kind: $U_{d}(x) = C^{(1)}_d(x)$.

Stieltjes [29] (see Theorem 6.21.1 of [28]) established the following relation between the zeros of the Chebyshev and Legendre polynomials.

#### Theorem 4.22

Let $\alpha _1> \alpha _2> \cdots > \alpha _{\lfloor d/2 \rfloor }$ be the positive roots of $U_{d}(x)$ and let $\beta _1> \beta _2> \cdots > \beta _{\lfloor d/2 \rfloor }$ be the positive roots of $P_{d}(x)$. Then, $\alpha _i < \beta _i$ for all *i*.

The relationship between the asymmetric additive convolution and the Chebyshev polynomials will make use of the generating function (25). To aid in this endeavor, we recall the following well-known generalization of the *binomial theorem* (see, for example, [30]).

#### Theorem 4.23

The function $(1+z)^{-k}$ has the formal power series expansion

$$\begin{aligned} \frac{1}{(1+z)^k} = \sum _{i=0}^{\infty } \left( {\begin{array}{c}k+i-1\\ i\end{array}}\right) (-z)^i. \end{aligned}$$

#### Lemma 4.24

For $d \ge 0$ and positive $\lambda$ and $\mu$,

#### Proof

By (25), we have

$$\begin{aligned} \sum _{d} U_d(x)~t^d = \frac{1}{1 - 2 xt + t^2} \end{aligned}$$

and so

$$\begin{aligned} \sum _{d} \sqrt{\lambda \mu }^d U_d \left( \frac{x - \lambda - \mu }{2 \sqrt{\lambda \mu }}\right) ~t^d&= \frac{1}{1 - (x - \lambda - \mu )t + \lambda \mu t^2} \\ {}&= \frac{1}{( 1- \lambda t)(1 - \mu t) - x t} \\ {}&= \frac{1}{( 1- \lambda t)(1 - \mu t)}\frac{1}{1 - \frac{x t}{(1 -\lambda t)(1 - \mu t)}} \\ {}&= \sum _{k \ge 0} \frac{x^{k} t^k}{(1-\lambda t)^{k+1}(1-\mu t)^{k+1}} \\ {}&= \sum _{i, j, k \ge 0} \left( {\begin{array}{c}k+i\\ i\end{array}}\right) \left( {\begin{array}{c}k+j\\ j\end{array}}\right) x^{k}(-\lambda )^i(-\mu )^j t^{i+j+k} \end{aligned}$$

so the coefficient of $t^d$ can be written (setting $\ell = d - k$)

$$\begin{aligned} \sum _{\ell =0}^d x^{d-\ell } \sum _{i+j = \ell }\left( {\begin{array}{c}d-j\\ i\end{array}}\right) \left( {\begin{array}{c}d - i\\ j\end{array}}\right) (-\lambda )^i(-\mu )^j. \end{aligned}$$

On the other hand, the formula for the asymmetric convolution also gives us$\square$

The relationship between the symmetric additive convolution and the Legendre polynomials can be established by applying Theorem 2.11

#### Lemma 4.25

For $d \ge 0$ and positive $\lambda$ and $\mu$,

#### Proof

We start by recalling one of the well-known formulas for Legendre polynomials [28]:

$$\begin{aligned} P_d(x) = \frac{1}{2^d} \sum _i \left( {\begin{array}{c}d\\ i\end{array}}\right) ^2 (x-1)^i (x+1)^{d-i}. \end{aligned}$$

On the one hand we have

$$\begin{aligned} P_d\left( \frac{x^2 - \lambda - \mu }{2 \sqrt{\lambda \mu }} \right)&= \frac{1}{2^d} \sum _i \left( {\begin{array}{c}d\\ i\end{array}}\right) ^2 \left( \frac{x^2 - \lambda - \mu }{2 \sqrt{\lambda \mu }} - 1 \right) ^i \left( \frac{x^2 - \lambda - \mu }{2 \sqrt{\lambda \mu }} + 1\right) ^{d-i} \\ {}&= \frac{1}{(4 \sqrt{\lambda \mu })^d} \sum _i \left( {\begin{array}{c}d\\ i\end{array}}\right) ^2 (x^2 - (\sqrt{\lambda } + \sqrt{\mu })^2)^i (x^2 - (\sqrt{\lambda } - \sqrt{\mu })^2)^{d-i}. \end{aligned}$$

On the other hand, we can use Theorem 2.11 to calculate . There are four possible root sums that will appear: $\{ \pm (\sqrt{\lambda } + \sqrt{\mu }), \pm (\sqrt{\lambda } - \sqrt{\mu }) \}$. Furthermore, it is easy to check that

1. the $\pm (\sqrt{\lambda }+\sqrt{\mu })$ terms appear the same number of times in every pairing
2. the $\pm (\sqrt{\lambda }-\sqrt{\mu })$ terms appear the same number of times in every pairing
3. the probability of having *i* copies of $(\sqrt{\lambda }+\sqrt{\mu })$ and $d-i$ copies of $(\sqrt{\lambda }-\sqrt{\mu })$ is $$\left( {\begin{array}{c}d\\ i\end{array}}\right) ^2/\left( {\begin{array}{c}2d\\ d\end{array}}\right) $$

Hence we have$\square$

#### Proof of Lemma 4.21

We prove this in the case that *d* is even. The proof with *d* odd is similar, except that there is one extra common term for the root at 0. Let $1> \alpha _1> \alpha _2> \cdots > \alpha _{ d/2 }$ be the positive roots of $U_{d}(x)$ and let $\alpha _{d+1-i} = -\alpha _i$ be the negative roots. Similarly, let $1> \beta _1> \beta _2> \cdots > \beta _{d}$ be the roots of $P_{d}(x)$. As $(\lambda + \mu ) / 2 \sqrt{\lambda \mu } \ge 1$, the roots ofare

$$\begin{aligned} \pm \left( \sqrt{\lambda \mu } \beta _i + (\lambda + \mu ) \right) ^{1/2} \end{aligned}$$

and the roots ofare

$$\begin{aligned} \pm \left( \sqrt{\lambda \mu } \alpha _i + (\lambda + \mu ) \right) ^{1/2}. \end{aligned}$$

By Theorem 4.22 the largest root of the first polynomial is larger than the largest root of the second, and for every *t* larger than that root,where the last equality follows by reversing the previous reasoning. This implies thatwhere $w = 1/ \alpha d$. $\square$

## Declaration

### Declaration

The original version of this paper was posted to arXiv in 2015 as paper number 1504.00350. This version is a substantial revision of the original.
